# Molecular mechanics of coiled coils loaded in the shear geometry†
†Electronic supplementary information (ESI) available: CD measurements for the determination of secondary structure and the thermal stability of the coiled coils as well as additional results of the SMFS experiments and the SMD simulations are included in the supporting information. See DOI: 10.1039/c8sc01037d


**DOI:** 10.1039/c8sc01037d

**Published:** 2018-04-23

**Authors:** Melis Goktas, Chuanfu Luo, Ruby May A. Sullan, Ana E. Bergues-Pupo, Reinhard Lipowsky, Ana Vila Verde, Kerstin G. Blank

**Affiliations:** a Max Planck Institute of Colloids and Interfaces , Mechano(bio)chemistry , Science Park Potsdam-Golm , 14424 Potsdam , Germany . Email: Kerstin.Blank@mpikg.mpg.de; b Max Planck Institute of Colloids and Interfaces , Department of Theory & Bio-Systems , Science Park Potsdam-Golm , 14424 Potsdam , Germany . Email: Ana.Vilaverde@mpikg.mpg.de

## Abstract

Coiled coils are important nanomechanical building blocks in biological and biomimetic materials. A mechanistic molecular understanding of their structural response to mechanical load is essential for elucidating their role in tissues and for utilizing and tuning these building blocks in materials applications. Using a combination of single-molecule force spectroscopy (SMFS) and steered molecular dynamics (SMD) simulations, we have investigated the mechanics of synthetic heterodimeric coiled coils of different length (3–4 heptads) when loaded in shear geometry. Upon shearing, we observe an initial rise in the force, which is followed by a constant force plateau and ultimately strand separation. The force required for strand separation depends on the coiled coil length and the applied loading rate, suggesting that coiled coil shearing occurs out of equilibrium. This out-of-equilibrium behaviour is determined by a complex structural response which involves helix uncoiling, uncoiling-assisted sliding of the helices relative to each other in the direction of the applied force as well as uncoiling-assisted dissociation perpendicular to the force axis. These processes follow a hierarchy of timescales with helix uncoiling being faster than sliding and sliding being faster than dissociation. In SMFS experiments, strand separation is dominated by uncoiling-assisted dissociation and occurs at forces between 25–45 pN for the shortest 3-heptad coiled coil and between 35–50 pN for the longest 4-heptad coiled coil. These values are highly similar to the forces required for shearing apart short double-stranded DNA oligonucleotides, reinforcing the potential role of coiled coils as nanomechanical building blocks in applications where protein-based structures are desired.

## Abstract 2

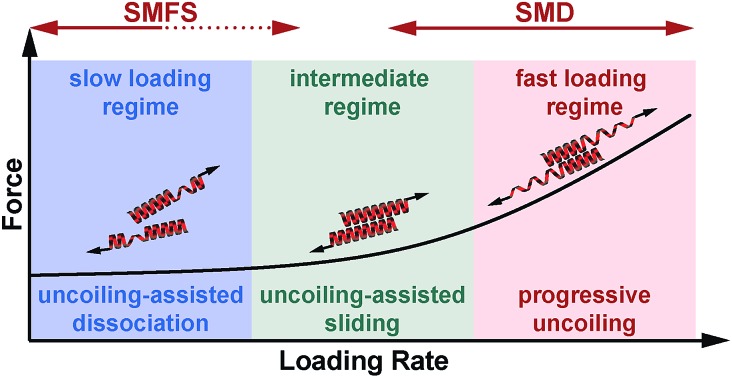
Shearing of short, dimeric coiled coils proceeds *via* three competing timescale-dependent mechanisms: progressive helix uncoiling, uncoiling-assisted sliding and dissociation.

## Introduction

**Fig. 1:**
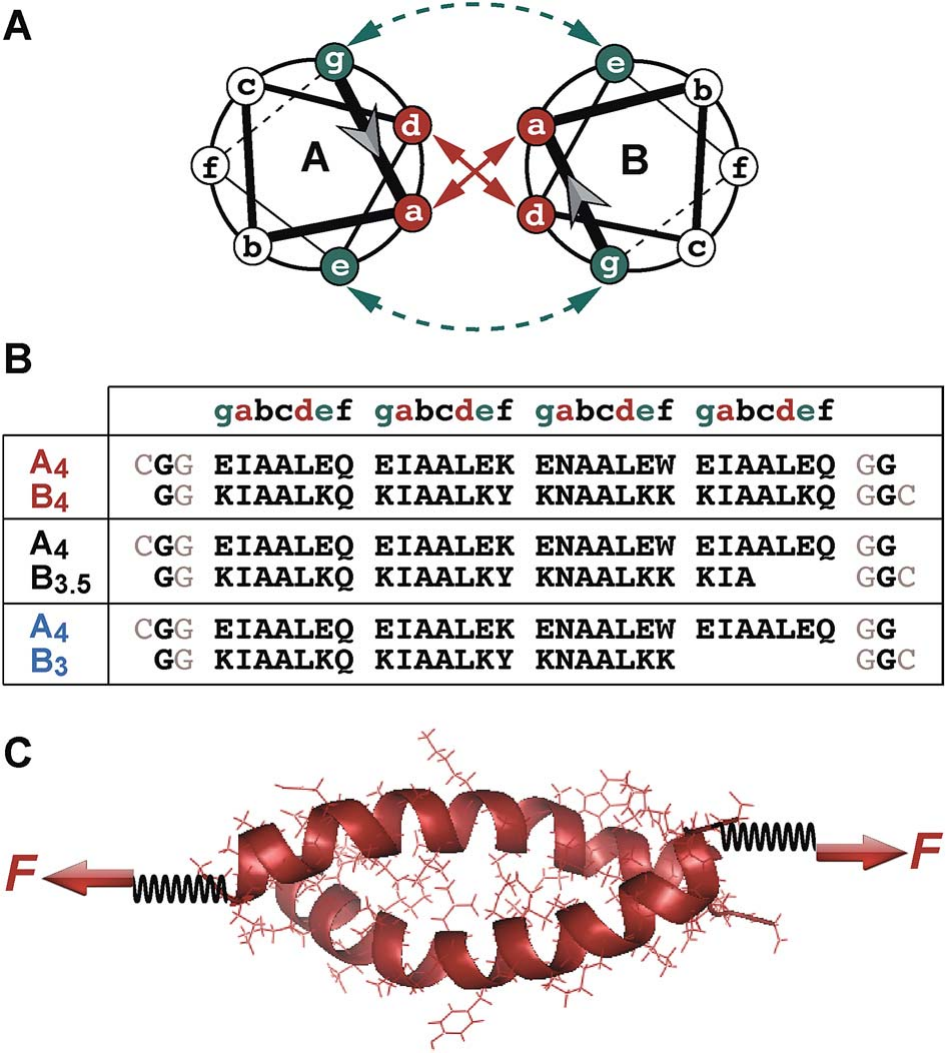
Experimental setup. (A) Structure of a heterodimeric coiled coil in the helical wheel representation. (B) Sequences of the coiled coil heterodimers used. The sequences used in the SMFS experiments contained two glycine residues at each terminus as well as a cysteine residue for site-specific immobilization. Only one glycine and no cysteine was present in the sequences used for the SMD simulations. (C) Geometry of force application for studying the mechanical stability of coiled coils under shear loading. The force was applied at the N-terminus of peptide A_4_ and at the C-termini of peptides B_4_, B_3.5_ and B_3_, utilizing cysteine residues introduced at the respective termini. In the SMFS experiments, the spring represents a poly(ethylene glycol) linker, which was used to couple the individual peptides to the surface and the AFM cantilever. In the SMD simulations, two virtual harmonic springs were introduced. The distal end of the spring present at the N-terminus of A_4_ was fixed, whereas the distal end of the spring attached at the C-terminus of the B peptides was displaced parallel to the helical axis at a constant speed. The initial structure of each coiled coil was produced using Avogadro^48^ and equilibrated before the SMD simulations.

Coiled coils are widespread structural motifs, which occur in a large variety of proteins; approximately 10% of all eukaryotic proteins contain coiled coil domains.^1^ The structurally simplest coiled coil consists of two α-helices wrapped around each other to form a superhelical assembly.^2^–^6^ Each α-helix is composed of a repetitive pattern of seven amino acids, (*abcdefg*)_*n*_, called a heptad repeat (Fig. 1A and B). This specific heptad pattern drives the folding and dimerization of the helices, whereby positions *a* and *d* are often occupied by hydrophobic residues. These residues form the dimer interface, which is key for stabilizing the coiled coil structure. Salt bridges, formed between oppositely charged residues at *e* and *g* positions, contribute additional stability. The solvent-exposed residues *b*, *c* and *f* are more variable, but are crucial for the stability of the individual α-helices.^7^

Coiled coil-containing proteins play a fundamental role in processes such as transcription, gene regulation, chromosome segregation, membrane fusion, muscle contraction, blood clotting and molecular transport.^4^,^5^ In addition, many structural proteins in the cytoskeleton and in the extracellular matrix possess coiled coil structures.^4^,^8^ Examples of cytoskeletal proteins are the intermediate filament proteins vimentin,^9^–^12^ desmin^13^ and keratin,^8^ as well as the molecular motor proteins myosin,^14^–^16^ kinesin^17^ and dynein.^18^,^19^ Fibrin,^20^–^22^ tenascin^23^ and laminin^24^ represent examples of coiled coil-containing proteins on the extracellular side. The widespread occurrence of coiled coils in mechanically active, as well as in structural proteins, is clear evidence that the molecular function of these proteins crucially depends on the mechanics of their coiled coil building blocks.

With the goal of shedding light on the molecular response of coiled coil structures to an externally applied force, single-molecule force spectroscopy (SMFS) and molecular dynamics (MD) were initially used to investigate a small number of natural coiled coil-containing proteins, such as myosin,^14^–^16^ vimentin^9^–^12^ and fibrin.^20^–^22^ When the force was applied parallel to the helical axis, these structures showed a universal, 3-phase response to the applied force.^9^,^10^,^12^,^14^–^16^,^21^,^22^,^25^,^26^ In phase I, an almost linear rise in the force was observed upon stretching the coiled coil. This increase in force originates from extending the coiled coil against entropic forces and from mechanically loading intrahelical hydrogen bonds. At 10–25% strain the force remained constant and a long force plateau was observed. During this plateau phase (phase II) intrahelical hydrogen bonds are continuously breaking and the individual helices uncoil at an almost constant force. In many cases the uncoiled structure is stabilized by interstrand hydrogen bonds and a β-sheet structure is formed (α–β transition).^25^ At strains larger than 80%, the force rises steeply (phase III), representing the stretching of possible β-sheet structures.^26^,^27^ As the coiled coils investigated so far differ in sequence and oligomerization state, the universality of this behaviour suggests that helix uncoiling represents a fundamental mechanistic response of coiled coils to an applied force. This response has been compared with the well-known overstretching transition of DNA,^14^,^28^–^36^ which is characterized by a force plateau at approximately 65 pN. For the coiled coils tested experimentally, the plateau was observed at forces between 20–60 pN.^14^–^16^,^21^ As full-length proteins were used in these initial experiments, which were anchored to the force transducer non-specifically, it was not clearly defined which portions of the structure were stretched under the applied force. Furthermore, the exact geometry of force application is unknown, as the attachment sites on the superhelix were random.^30^

To overcome these limitations and to investigate the sequence–structure–mechanics relationship of coiled coils in more systematic detail, site-specific coupling strategies were used in more recent experiments so that the mechanical coordinate was precisely defined. Using coiled coil homodimers, the force was applied to both helices at the same terminus so that the coiled coils were mechanically loaded in the so-called ‘unzip’ geometry.^11^,^17^,^37^,^38^ In this geometry, the mechanical unfolding of coiled coils, such as the GCN4 leucine zipper^37^,^38^ and vimentin,^11^ showed that the structures unfold at forces between 8–15 pN. Using coiled coils of different length, it was shown that unzipping is characterized by the sequential uncoiling of helical turns and that the unzipping force weakly depends on coiled coil sequence, but not on coiled coil length.^37^ At slow pulling speeds uncoiling was fully reversible, suggesting that mechanical coiled coil unfolding and refolding occurs at equilibrium. At faster pulling velocities, hysteresis was observed in the initial stages of refolding. This hysteresis was assigned to the formation of a helical seed, which is required before helix formation can propagate at a high rate.^17^ It is worth noting that the unzipping behaviour and even the unzipping forces are highly similar to what has been observed for DNA.^30^,^39^ This similarity strongly suggests that coiled coils may serve as equally powerful nanomechanical building blocks in a large number of applications.

Synthetic coiled coils are already being used as building blocks for the development of protein-based nanostructures.^40^–^43^ Moreover, they find application as dynamic crosslinks in biomimetic materials.^5^,^44^,^45^ To date, little attention has been paid to the mechanical stability of the coiled coil building blocks and the geometry of force application has not been considered as a design parameter. Here, we use a set of short heterodimeric coiled coils with lengths of 3–4 heptads to investigate their response when mechanically loaded in shear geometry, a mode which has not yet been characterized experimentally. In contrast, the response of DNA structures to shear forces is well characterized^31^,^46^ and has been extensively compared with DNA unzipping. We use atomic force microscope (AFM)-based single molecule force spectroscopy (SMFS) to experimentally determine the mechanical stability of these coiled coils and steered molecular dynamics (SMD) simulations to gain insights into their structural response to the applied force. Our results show that the mechanical stability of coiled coils against shearing depends on the length of the superhelical structure as well as on the rate of the applied force (*i.e.* the loading rate), suggesting that coiled coil shearing occurs out of equilibrium. We further show that shearing is a complex process, which includes contributions from progressive helix uncoiling, uncoiling-assisted sliding as well as dissociation of partially uncoiled helices. Most importantly, all shear forces measured are higher than previously measured unzipping forces,^11^,^17^,^37^,^38^ revealing that coiled coils loaded in different geometries do exhibit different mechanical stabilities.

## Results

### Experimental design

The coiled coil model system used in this work is based on a *de novo* designed set of short heterodimeric coiled coils, recently introduced by Woolfson *et al.*^47^ These sequences have been chosen as they are based on a highly regular (IAALXXX)_*n*_ repeat pattern (Fig. 1A and B), which results in a high thermodynamic stability. The 4-heptad coiled coil (CC-A_4_B_4_) possesses a dissociation constant *K*_D_ smaller than 10^–10^ M and a melting temperature above 80 °C. Truncated sequences, where 1 or 2 hydrophobic contacts were consecutively deleted at the C-terminus of one helix, still possess melting temperatures of 61 °C (CC-A_4_B_3.5_) and 39 °C (CC-A_4_B_3_) (Fig. S1 and S2, Table S1†). Most importantly, this set of sequences has originally been optimized to increase the specificity of heterodimer formation. Heterospecificity and helix orientation were mostly guided by oppositely charged lysine and glutamic acid residues at the *e* and *g* positions. Furthermore, isoleucine in the third heptad was replaced by asparagine in each helix.^3^ The polar asparagine residues create a local hydrophilic region in the hydrophobic dimer interface, thereby destabilizing undesired structures, such as out-of-register helical arrangements or thermodynamically weak homodimers.

The sequences chosen are sufficiently short for allowing their preparation with solid phase peptide synthesis. In this way, both coiled coil-forming peptides can be handled and immobilized independently, which is essential for setting up SMFS experiments (Fig. 1C). To apply force to these structures in the shear geometry, the attachment points were located at the N-terminus of A_4_ and at the C-terminus of peptides B_4_, B_3.5_ and B_3_. CC-A_4_B_3.5_ and CC-A_4_B_3_ possess a C-terminal overhang of the A_4_ peptide, which is not able to fold into a helical structure as it lacks stabilizing interactions with the B peptides. For these truncated coiled coils the attachment sites were chosen such that the force directly acts on the helical part of the structure and does not travel through the overhanging part of A_4_ (Fig. 1B). For coupling to the AFM cantilever and the surface, cysteine residues were introduced at the respective termini to allow for the site-specific coupling of each peptide.^49^ Identical attachment sites were used for the SMD simulations to mimic the experimental setup as closely as possible, with the only difference that no cysteine and only one glycine was used at each terminus (Fig. 1B).

### Length dependence of coiled coil rupture determined with dynamic single-molecule force spectroscopy (SMFS)

To analyse the mechanical stability of these heterodimeric coiled coils, the A_4_ peptide was immobilized onto an amino-functionalized glass slide *via* a hetero-bifunctional NHS-PEG-maleimide spacer.^49^ The B_4_, B_3.5_ and B_3_ peptides were immobilized to amino-functionalized cantilevers, using the same PEG spacer. The surface, functionalized with the A_4_ peptide, was approached with the tip of the cantilever to allow the coiled coil to form. Subsequently, the cantilever was retracted from the surface at a constant speed and the coiled coil was loaded with an increasing force until it ruptured and the cantilever relaxed back to its equilibrium position.

**Fig. 2:**
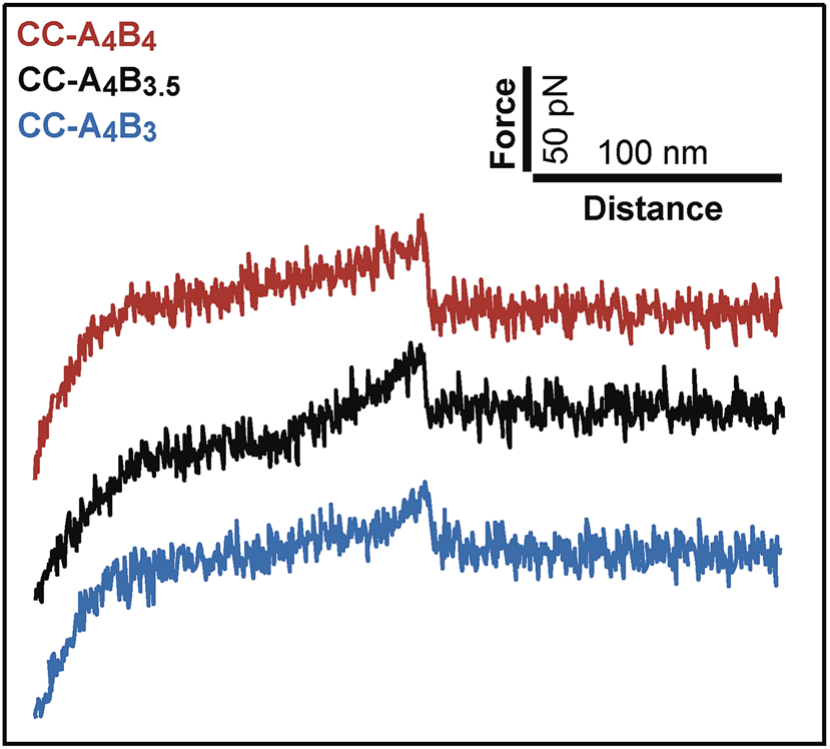
Representative force–extension curves of the different coiled coils measured with AFM-based single-molecule force spectroscopy. The force–extension curves show coiled coil rupture recorded at a retract speed of 400 nm s^–1^.

**Fig. 3:**
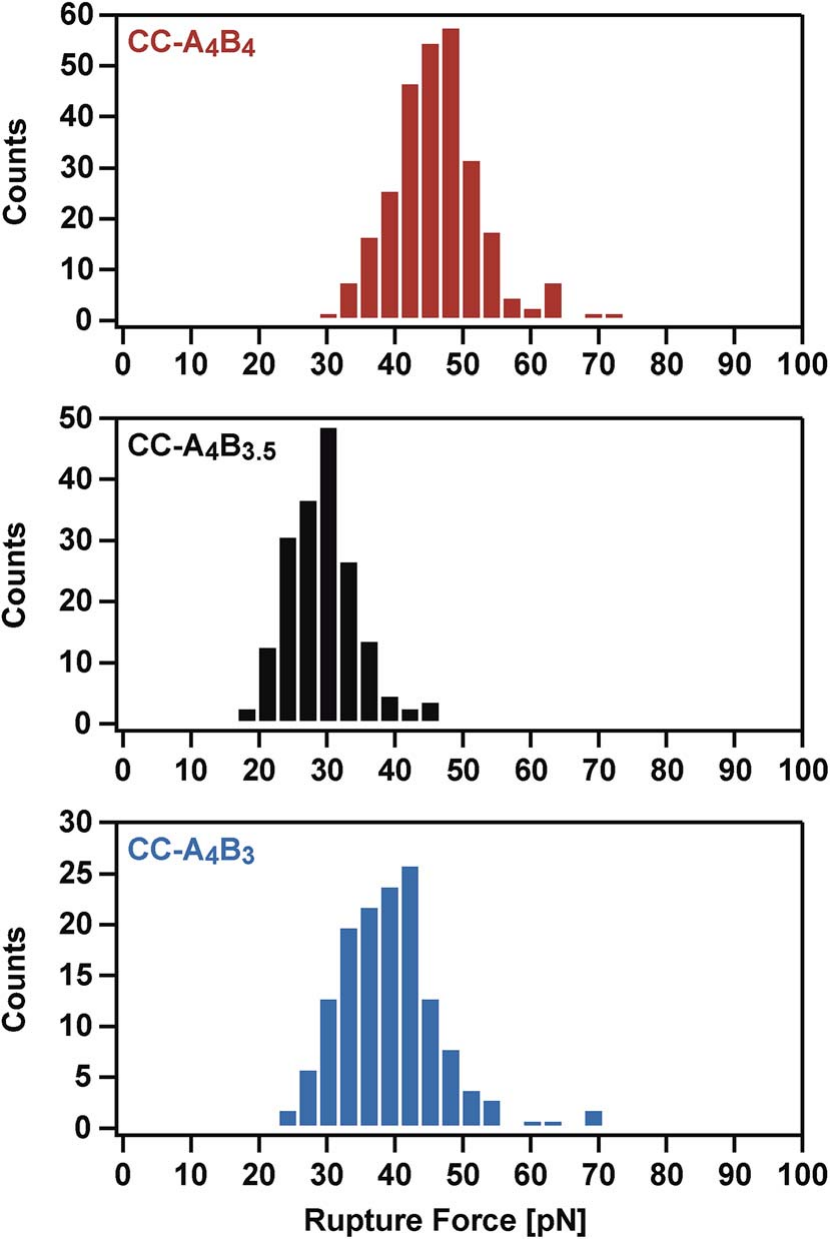
Representative rupture force histograms of the different coiled coils recorded at a retract speed of 400 nm s^–1^. The rupture force histograms contain 285 rupture events for CC-A_4_B_4_, 187 rupture events for CC-A_4_B_3.5_ and 145 rupture events for CC-A_4_B_3_, respectively.

Fig. 2 shows typical force–extension curves recorded for the three different coiled coils. For each of the coiled coils, dynamic SMFS was performed, recording several hundreds of force–extension curves with three different cantilevers. When comparing the rupture force histograms measured at a retract speed of 400 nm s^–1^, clear differences were observed for the three different coiled coils (Fig. 3). Using a Gaussian fit, which is frequently used to determine the most probable rupture force, values of 44 pN (CC-A_4_B_4_), 27 pN (CC-A_4_B_3.5_) and 37 pN (CC-A_4_B_3_) were obtained. These results already provide evidence that the rupture forces for coiled coil shearing are length-dependent and are higher than for coiled coil unzipping, which typically occurs at forces between 8–15 pN.^11^,^17^,^37^,^38^

**Fig. 4:**
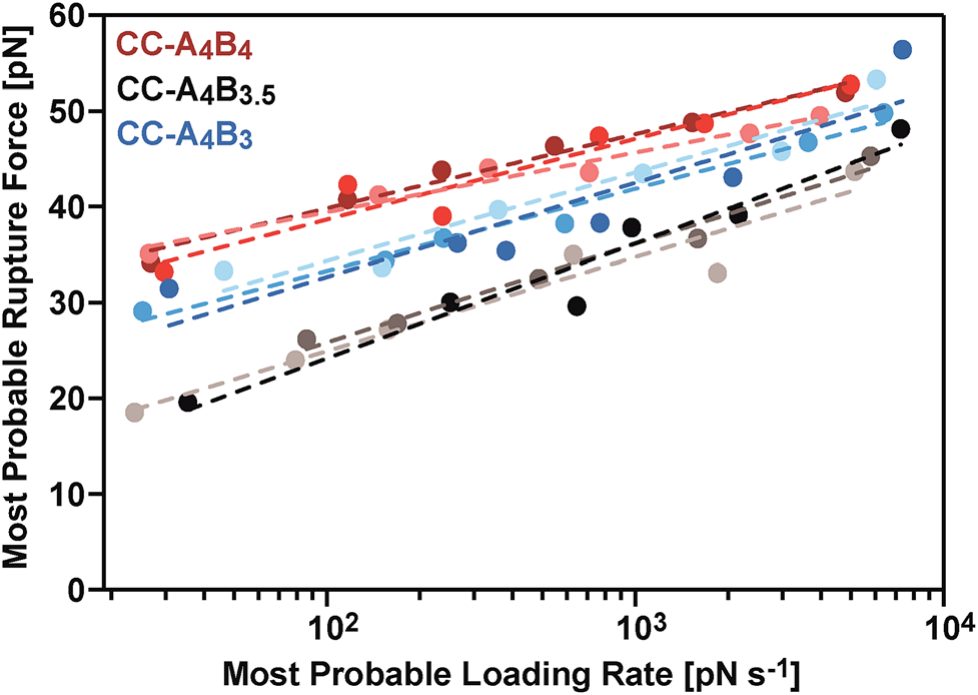
Bell–Evans plot showing a linear relationship between the most probable rupture forces and the logarithm of the corresponding loading rates. The lines represent fits to the Bell–Evans model to extract the *k*_off_SMFS_ and Δ*x*_SMFS_ values. For each coiled coil three different measurements with different cantilevers and surfaces were performed and analysed independently; the corresponding data are shown as different shades of the same colour.

The most probable rupture forces, obtained at different cantilever retract speeds (Fig. S3–S5†), were subsequently plotted against the corresponding most probable loading rates. Because of the non-linear stiffness of the PEG spacer, the loading rates ** = d*F*/d*t* were determined for every individual force–extension curve (slope *k*_S_ of the force–extension curve at the point of rupture, multiplied by the retract speed). The most probable loading rates were then obtained from their corresponding histograms (Fig. S3–S5†) and range from approximately 20 pN s^–1^ to 7500 pN s^–1^. The resulting *F vs.* ln ** plots (Fig. 4) demonstrate that the coiled coil rupture forces increase linearly with the logarithm of the loading rates, as predicted by the Bell–Evans model.^50^ Fitting the data to this model yields the extrapolated force-free dissociation rates *k*_off_SMFS_ and the corresponding potential widths Δ*x*_SMFS_ (Table 1). A comparison of the longest (CC-A_4_B_4_) and the shortest coiled coil (CC-A_4_B_3_) shows that the longest coiled coil possesses the slowest dissociation rate, *k*_off_SMFS_ = 3.2 × 10^–4^ s^–1^, and the largest potential width, Δ*x*_SMFS_ = 1.29 nm. CC-A_4_B_3_ shows a faster dissociation rate of 6.5 × 10^–3^ s^–1^ and a smaller potential width of 1.03 nm.

**Table 1:** Summary of the *k*_off_SMFS_ and Δ*x*_SMFS_ values for the different coiled coils obtained from Bell–Evans fits to the SMFS data. The values are the mean of three experiments performed with three different cantilevers and surfaces. The error represents the standard error of the mean

Heterodimer	*k*_off_SMFS_ [s^–1^]	Δ*x*_SMFS_ [nm]
CC-A_4_B_4_	(3.2 ± 2.1) × 10^–4^	1.29 ± 0.12
CC-A_4_B_3.5_	(1.1 ± 0.4) × 10^–1^	0.89 ± 0.05
CC-A_4_B_3_	(6.5 ± 2.4) × 10^–3^	1.03 ± 0.04

This trend in the dissociation rates is expected, considering the differences in the thermodynamic stability of these coiled coils. We tentatively interpret this trend in the following way: the coiled coil is deformed in the direction of the externally applied force, whereby the amount of stably folded structure is reduced. It is known that a minimum coiled coil length is required for maintaining a thermodynamically and kinetically stable structure.^37^,^51^ Once the mechanical deformation of the coiled coil has reached a critical magnitude, the remaining structure possesses a lower binding free energy and the probability for thermally-assisted dissociation perpendicular to the force axis increases. Clearly, for shorter heterodimers this instability already appears at smaller extensions, also explaining the observed correlation between the potential width and the coiled coil length. CC-A_4_B_3.5_, which contains an incomplete heptad repeat, is the mechanically weakest structure even though its thermodynamic stability was determined to be higher than the stability of CC-A_4_B_3_ (Table S1†). CC-A_4_B_3.5_ is characterized by the highest dissociation rate, *k*_off_SMFS_ = 1.1 × 10^–1^ s^–1^ and the smallest potential width, Δ*x*_SMFS_ = 0.89 nm, suggesting that the presence of incomplete heptads leads to a mechanical destabilization of the coiled coil.

### Steered molecular dynamics (SMD) simulations at different retract speeds

**Fig. 5:**
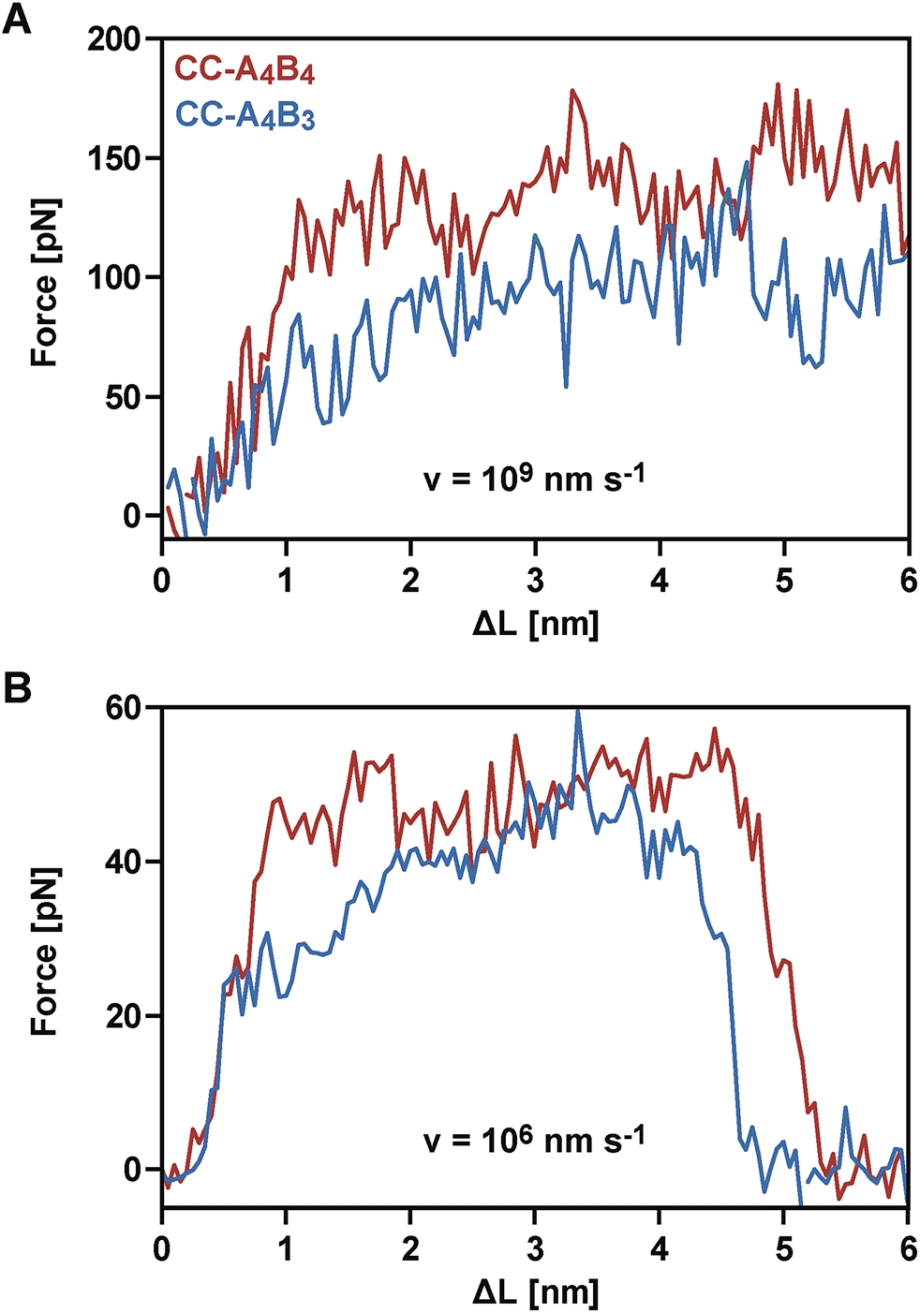
Averaged force–extension curves of the different coiled coils obtained from SMD simulations (*T* = 300 K; implicit solvent). The graph shows the forces as a function of extension (Δ*L* = *v* × *t*, where *v* is the retract speed and *t* is time). (A) Force–extension behaviour at the fastest retract speed (*v* = 10^9^ nm s^–1^). The results represent averages over 40 (CC-A_4_B_4_) and 20 (CC-A_4_B_3_) independent runs. (B) Force–extension behaviour at the slowest retract speed (*v* = 10^6^ nm s^–1^). The results represent averages over 5 (CC-A_4_B_3_) or 6 (CC-A_4_B_4_) independent runs.

To obtain a molecular understanding of the general mechanism of coiled coil shearing, we carried out SMD simulations for the two coiled coils with complete heptad repeats (CC-A_4_B_4_ and CC-A_4_B_3_), using the same sequences and pulling geometries as used in the SMFS experiments (Fig. 1). The only difference was that only one glycine and no cysteine residue were used at the N- and C-termini of the respective sequences. In the SMD simulations, two virtual harmonic springs replaced the PEG spacers used in the experiment. Pulling at the distal end of the virtual spring, the C-terminus of the B peptides was displaced parallel to the helical axis at a constant speed. The distal end of the N-terminal spring attached to the A_4_ peptide was fixed. To be able to simulate the coiled coils at the slowest retract speeds computationally possible, we first investigated if implicit solvent simulations capture the essential features of the coiled coil response to the applied shear force. For this purpose, initial simulations were performed with CC-A_4_B_4_, using an explicit and an implicit water model. The absolute force values obtained with the explicit water model are approximately 20% smaller than those seen for the implicit water model (Fig. 5 and S6†). Despite these quantitative differences, the two methods yield highly similar force–extension curves at all retract speeds tested, indicating that the simulations using an implicit water model are not unduly biased.

We therefore continued with the computationally less expensive implicit solvent simulations, using different retract speeds ranging from *v* = 10^6^ nm s^–1^ to *v* = 10^9^ nm s^–1^. The slowest retract speed is approximately 10–100 times lower than what has previously been used for the simulation of coiled coils.^22^,^26^,^27^ At all retract speeds, a very similar behaviour was observed for the two different coiled coils. The force–extension curves are characterized by an initial rise in the force (equivalent to phase I), followed by a plateau (Fig. 5). The transition into the plateau phase (phase II) occurs at a strain of 15–25% (Fig. S7†). The behaviour of these short, synthetic coiled coils is therefore highly similar to experimental and simulation results obtained earlier for long, natural coiled coils stretched parallel to the helical axis,^9^,^10^,^12^,^14^–^16^,^21^,^22^,^25^,^26^ even though phase III is absent. This absence is a direct result of the coiled coil length and the attachment geometry, which causes the individual strands to separate before phase III is reached. Strand separation occurs *via* the relative translation of the individual peptides in the direction of the applied force (see Movie 1 for *v* = 10^9^ nm s^–1^ and Movie 2 for *v* = 10^6^ nm s^–1^†). A highly similar force–extension behaviour was observed for a longer coiled coil, where the first two heptads of the N-terminus of CC-A_4_B_4_ were repeated, resulting in CC-A_6_B_6_ (Fig. S8† and accompanying text). Based on this simulation of CC-A_6_B_6_, performed at a retract speed of *v* = 10^7^ nm s^–1^, we concluded that investigating CC-A_4_B_3_ and CC-A_4_B_4_ is sufficient to describe the structural response of this coiled coil model system to shear forces.

**Fig. 6:**
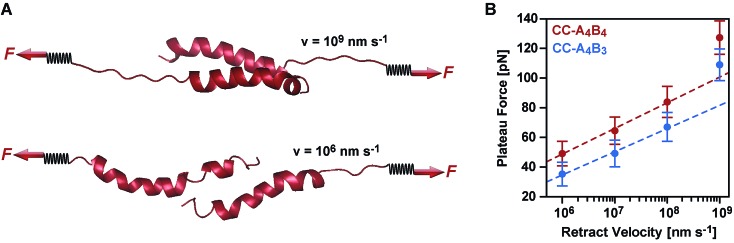
Coiled coil response to an applied shear force in SMD simulations. (A) Simulation snapshots of CC-A_4_B_4_ obtained at the fastest (*v* = 10^9^ nm s^–1^) and slowest (*v* = 10^6^ nm s^–1^) retract speeds. (B) Bell–Evans plot showing the relationship between the average plateau force and the corresponding retract speed. The plateau forces shown are calculated as the mean of the average plateau forces obtained in 5 (CC-A_4_B_3_) or 6 (CC-A_4_B_4_) simulation runs at *v* = 10^6^ nm s^–1^ and *v* = 10^7^ nm s^–1^, 20 runs for both coiled coils at *v* = 10^8^ nm s^–1^ and for CC-A_4_B_3_ at 10^9^ nm s^–1^, and 40 runs for CC-A_4_B_4_ at *v* = 10^9^ nm s^–1^. The error bars are the standard error of the mean. The average plateau force in each simulation run is calculated by averaging over an extension interval of [2 < Δ*L* < 6] nm for the two largest retract speeds, and [2 < Δ*L* < 4] nm otherwise. The lines represent fits to the Bell–Evans equation, using *k*_L_ = 40 pN nm^–1^ for converting the retract speed into loading rate. The data points obtained at the fastest retract speed of *v* = 10^9^ nm s^–1^ (corresponding to a loading rate of 4 × 10^10^ pN s^–1^) were not included into the Bell–Evans fit. At this retract speed, the response mechanism is dominated by progressive uncoiling instead of uncoiling-assisted sliding.

To obtain more detailed structural insight into the strand separation mechanism, we examined the evolution of the helical secondary structure in the simulation trajectories. The trajectories show that the mechanism of strand separation differs at the fastest and slowest retract speeds used (Fig. 6A). At the fastest retract speed (*v* = 10^9^ nm s^–1^) the helices begin to uncoil at the points of force application. When extended further, uncoiling propagates along the helices until all helical structure is lost and the strands separate. The propagation of helix uncoiling from the points of force application has also been observed in other simulations of dimeric and trimeric coiled coils that were mechanically loaded parallel to their helical axis.^9^,^15^,^26^,^52^,^53^ In contrast, the helices seem to slide against each other at the slowest retract speed used (*v* = 10^6^ nm s^–1^). A detailed inspection of the secondary structure and the interhelical contacts (hydrophobic interactions and salt bridges; Fig. S9†) indicates that the helices do uncoil in response to the applied force, but are able to recoil during the timescale of the simulation. We term this mechanism, which facilitates a relative displacement of the helices against each other, uncoiling-assisted sliding. At intermediate retract speeds, the extension of the coiled coils involves a combination of both mechanisms. Helix recoiling has also been observed in constant-force simulations of dimeric coiled coils loaded in a tensile geometry, *i.e.* where all termini were loaded simultaneously. In these simulations, helix recoiling occurred with a much higher probability at lower loads.^54^

Extrapolating from simulation data obtained at retract speeds faster than 10^9^ nm s^–1^, Buehler *et al.*^9^,^10^ predicted that stretching of the vimentin coiled coil in a tensile geometry involves a change in mechanism at a retract speed of approx. 10^8^ nm s^–1^. This speed falls into the range used in our simulations. For fast retract speeds, Buehler *et al.*^9^,^10^ observe that only hydrogen bonds next to the point of force application feel the force and that helix uncoiling is highly localized, as we also observe in our simulation at the fastest retract speed of 10^9^ nm s^–1^ (Fig. 6A). For retract speeds slower than 10^8^ nm s^–1^, it is proposed that the force is distributed more homogeneously throughout the structure so that uncoiling can initiate anywhere in the helices. This regime matches the timescales observed for the formation of helical structure in individual helices^55^,^56^ and is reproduced in our simulations, which show that the helices dynamically uncoil and recoil in response to the applied force. Whereas Buehler *et al.*^9^,^10^ propose that helix uncoiling involves full helical turns (3–4 amino acids), our simulations show that also smaller numbers of amino acids uncoil and recoil in response to the applied force (Fig. S10†). Overall, these observations suggest a retract speed dependent mechanism, with an increasing contribution of helix recoiling and uncoiling-assisted sliding at slower retract speeds.

### Comparison of SMFS experiments and SMD simulations

Following this mechanistic interpretation of coiled coil shearing in the SMD simulations, our next goal was to quantitatively compare the force range seen in the SMD simulations with the data obtained from the SMFS experiments. The force plateau observed in the simulations shows a maximum length of 4 nm for the longest coiled coil CC-A_4_B_4_. Considering the thermal noise level present in the experimental force–extension curves (Fig. 2), the possible occurrence of a short force plateau directly before the rupture event can most likely not be resolved. The possible contribution of helix uncoiling and uncoiling-assisted sliding to coiled coil deformation and strand separation in the SMFS experiments can therefore not be determined directly from the force–extension curves. Instead, we compare the loading rate dependence of the experimentally determined rupture forces and of the plateau forces (phase II) observed in the simulations.

The SMD force–extension curves of CC-A_4_B_4_ and CC-A_4_B_3_ show that the transition from phase I to phase II occurs at different forces (Fig. 5). In addition, the plateau forces increase with faster retract speeds. The plateau forces were extracted for both coiled coils and the corresponding retract speeds were converted into loading rates by estimating the proportionality constant *k*_L_ between loading rates (d*F*/d*t*) and retract speeds (*v*): d*F*/d*t* = *k*_L_*v*. We can only obtain a crude estimate of *k*_L_ in the force–extension plots (Fig. 5): *k*_L_ = 40 pN nm^–1^. As the transition between phase I and phase II is clearest for CC-A_4_B_4_ at the slowest retract speed, we estimate *k*_L_ based on this simulation only, and assume that this estimate holds for both coiled coils studied. The phase I → II transition occurs at Δ*L* = 1 nm for CC-A_4_B_4_. The SMD loading rates thus vary between 4 × 10^7^ pN s^–1^ and 4 × 10^10^ pN s^–1^. The slowest loading rate in the simulations is therefore still three orders of magnitude larger than the fastest experimentally accessible one. Despite this difference in loading rates, the plateau forces (35–85 pN) observed at the three slowest loading rates are highly similar to the experimentally determined rupture forces (20–50 pN) (Fig. 4 and 6B). Only the rupture force value at the fastest loading rate tested in the SMD simulations exceeds 100 pN, which is a direct result of the loading rate (retract speed)-dependent strand separation mechanism discussed above.

The plateau forces are always larger for CC-A_4_B_4_ than for CC-A_4_B_3_. The average plateau force for CC-A_6_B_6_, simulated only at a retract speed of *v* = 10^7^ nm s^–1^, is (84 ± 1) pN (Fig. S8†), which is higher than for the other two coiled coils at the same retract speed. For both CC-A_4_B_4_ and CC-A_4_B_3_, a linear relationship exists between the average plateau force and the logarithm of the loading rate (Fig. 6B), analogous to experiment. Fitting the Bell–Evans model to the data estimates the corresponding force-free parameters *k*_off_SMD_ = 9 × 10^3^ s^–1^ and Δ*x*_SMD_ = 0.54 nm for CC-A_4_B_4_, and comparable values for CC-A_4_B_3_ (Table 2). The Δ*x*_SMD_ values are smaller than the Δ*x*_SMFS_ values. Whereas the Δ*x*_SMD_ values represent helix uncoiling and recoiling in phase II, the Δ*x*_SMFS_ values describe strand separation (*i.e.* rupture in the SMFS experiment). The result that the Δ*x*_SMFS_ are larger than the Δ*x*_SMD_ values directly suggests that strand separation occurs after the phase I → II transition where parts of the helical structure are already uncoiled. In addition, larger differences are observed between the Δ*x*_SMFS_ values of CC-A_4_B_4_ (1.29 nm) and CC-A_4_B_3_ (1.03 nm) when compared to the corresponding Δ*x*_SMD_ values. This confirms our earlier interpretation that longer coiled coils can tolerate larger deformations before the strands separate under experimental conditions.

**Table 2:** Summary of the *k*_off_SMD_ and Δ*x*_SMD_ values for the different coiled coils obtained from a Bell–Evans fit to the simulation data. The error represents the standard error of the parameters, obtained using a non-linear fit

Heterodimer	*k*_off_SMD_ [s^–1^]	Δ*x*_SMD_ [nm]
CC-A_4_B_4_	(9 ± 5) × 10^3^	0.54 ± 0.04
CC-A_4_B_3_	(4 ± 2) × 10^4^	0.59 ± 0.04

The *k*_off_SMD_ and *k*_off_SMFS_ values differ by several orders of magnitude. In the SMD simulations, strand separation occurs on a timescale that is 4–7 orders of magnitude faster than in the experiment. In addition, the *k*_off_SMD_ values for the two different coiled coils differ only by a factor of 4, whereas the *k*_off_SMFS_ values show larger differences. These differences between SMD and SMFS results suggest that the progressive uncoiling of helical turns and uncoiling-assisted sliding may not be the main process leading to strand separation in the SMFS experiments, even though they may contribute. As coiled coils of less than five hydrophobic contacts are not thermodynamically stable^37^,^51^ we suggest that coiled coil deformation yields intermediates with a reduced binding free energy, which can easily dissociate.

This interpretation is further supported when comparing the *k*_off_SMFS_ value of CC-A_4_B_4_ to the true thermal off-rate *k*_off_, determined in the absence of an applied force. For CC-A_4_B_4_, the equilibrium dissociation constant *K*_D_ was determined^47^ to be <10^–10^ M and the on-rates *k*_on_ for dimeric coiled coils of similar length are reported^57^,^58^ to lie in the range of 10^5^–10^6^ M^–1^ s^–1^, which implies *k*_off_ < 10^–4^ s^–1^. Even though it is not possible to obtain an exact *k*_off_ value for CC-A_4_B_4_, this simple calculation suggests that *k*_off_ is smaller than *k*_off_SMFS_. This is a clear hint that the applied force deforms the coiled coil and causes partial helix uncoiling, perhaps accompanied by sliding. In combination, both processes reduce the amount of folded coiled coil structure, thereby increasing the probability of thermally activated strand separation in directions perpendicular to the force axis. As the timescale of the thermally activated process is several orders of magnitude larger than the simulation timescale, this strand separation mechanism cannot be observed in the SMD simulations. Overall, these results suggest a third strand separation mechanism at experimentally accessible loading rates. In the following, we term this strand separation mechanism uncoiling-assisted dissociation.

The different *k*_off_ and Δ*x* values, determined in different loading rate regimes, do not only reveal the existence of different strand separation mechanisms, they also suggest a continuous transition between these different mechanisms when altering the loading rate. It is therefore critical to note that the *F vs.* ln ** plots are only linear in a small range of loading rates, where one mechanism is dominant. As a consequence, the *k*_off_ and Δ*x* values obtained from the Bell–Evans fits do not describe coiled coil dissociation in the absence of force. Extrapolation to force-free conditions would require a model that describes an unbinding process determined by at least two competing timescale-dependent mechanisms.

## Discussion

Considering the important role of coiled coils as structural building blocks in natural and synthetic molecular architectures, it is of fundamental importance to mechanistically understand the response of coiled coils to externally applied forces. Whereas a number of experimental and simulation studies have been performed where coiled coils were stretched parallel to the helical axis, this is the first report where the mechanical response of coiled coils to a defined shear force is observed experimentally. Making use of three structurally related coiled coils of different length, we show that coiled coil shearing is mechanistically complex, involving a dependence on both the applied strain and the loading rate. Just as for the tensile geometry, the initiation of helix uncoiling is observed at 15–25% strain (phase I → II transition; Fig. 5 and S7†). Even though this fundamental strain-dependent response appears to be present in both tensile and shear pulling geometries, crucial differences between the two geometries are also observed.

**Fig. 7:**
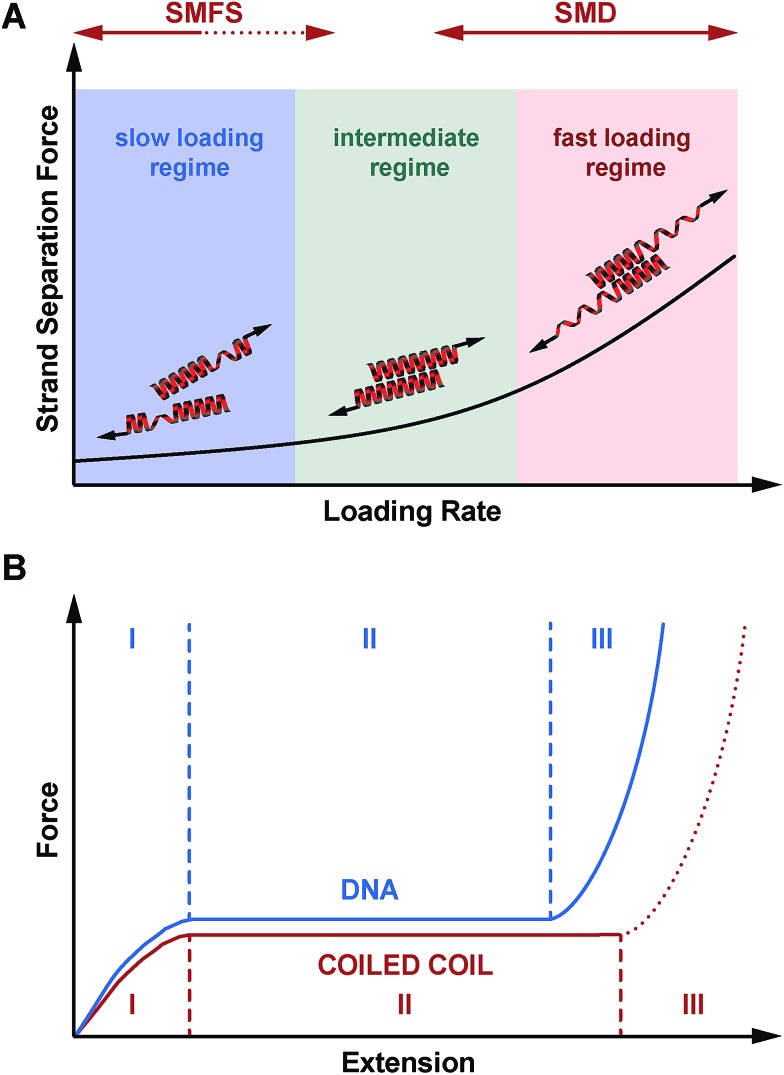
Mechanical response of coiled coils to shear forces. (A) Loading rate-dependent response of coiled coils mechanically loaded in shear geometry. In the fast deformation regime, accessible only in SMD simulations, helix uncoiling is initiated at the point of force application. Strand separation occurs *via* the propagation of helix uncoiling along the helical axis until all helical structure is lost. In the intermediate regime, where helix recoiling is possible, uncoiling-assisted sliding becomes the dominant mechanism. In the slow deformation regime, uncoiling-assisted dissociation perpendicular to the force axis is facilitated once deformation has reached a critical magnitude. (B) Comparison of mechanical DNA and coiled coil unfolding as a function of extension. When stretched parallel to the helical axis, coiled coils show a universal, 3-phase response similar to the DNA overstretching transition. Phase III is always expected to occur for tensile geometries, where strand separation is prohibited. For the shear geometry, the existence of phase III is expected to depend on the thermodynamic stability of the molecule, as dissociation perpendicular to the force axis is always possible at any extension. For the coiled coils investigated here, phase III is not reached and strand separation occurs in phase I or II.

In the tensile geometry, all termini are fixed and the coiled coil can only extend in the direction of the applied force. In contrast, the shear geometry allows a relative displacement of the coiled coil strands parallel to the helical axis. These additional degrees of freedom allow uncoiling-assisted sliding as well as uncoiling-assisted dissociation. These two strand-separation mechanisms compete with each other, with uncoiling-assisted dissociation being the dominant mechanism at experimentally relevant loading rates (Fig. 7A). It should be noted that structures with a different heptad register, as generated during sliding, are not stable for the coiled coils investigated here. The resulting structures would contain two asparagine–isoleucine pairings, which would destabilize the coiled coil thermodynamically and kinetically. In this context, we propose that strand separation arising from uncoiling-assisted sliding requires longer coiled coil sequences to contribute to strand separation at experimentally relevant timescales.

In general, it can be assumed that the relative probability of these two strand separation mechanisms does not only depend on the loading rate, but is also affected by the coiled coil length and sequence. Coiled coils with a highly repetitive sequence may more easily undergo uncoiling-assisted sliding as also different heptad registers are possible. In natural coiled coils, which have a less defined hydrophobic core and charge pattern, alternative heptad registers may lead to highly unstable structures so that uncoiling-assisted sliding may not be observable. As mentioned above, the short coiled coils studied here dissociate after the uncoiling of a relatively small amount of helical structure. Longer coiled coils can tolerate larger amounts of uncoiling, while still being thermodynamically stable. This will increase the probability of uncoiling-assisted sliding, provided that the sequence of the coiled coil allows alternative stable structures to exist. Interestingly, sliding has been proposed to be directly involved in the force transmission mechanism in the molecular motor protein dynein, which possesses a long dimeric coiled coil,^18^,^19^,^59^ and may also be involved in allosteric signal propagation in other coiled coil proteins.^60^ It appears likely, that the sequence of natural coiled coils is fine-tuned to balance these possible response mechanisms to an applied shear force.

Besides understanding the fundamental mechanism of coiled coil shearing, another key goal of this study was to compare the shear response to mechanical strand separation in the unzip geometry, as reported earlier for a number of natural coiled coil sequences of different length. Unzipping also shows a force plateau (8–15 pN),^11^,^17^,^37^,^38^ which appears to be almost independent of coiled coil length and overall sequence. The length independence suggests that the structure is unfolded in a turn-by-turn fashion and that strand separation occurs in equilibrium. Our results demonstrate that this is clearly not the case when the coiled coils are loaded in the shear geometry. In addition to a clear length dependence, the forces required for strand separation increase with the loading rate as predicted by the Bell–Evans model. This observed difference between the shear and unzip geometries is highly similar to the behaviour of short, double stranded DNA mechanically loaded in either the shear^31^,^46^ or unzip geometry, even though a direct comparison is difficult. DNA consists of two tightly bound, linear strands wound into a helix. In contrast, the individual coiled coil strands are helical and wrap around each other, forming a superhelix. Whereas DNA shearing proceeds *via* the rupture of interstrand base pairing interactions, coiled coils respond to the applied force by unfolding of individual helices, most likely before the first interstrand hydrophobic contacts and salt bridges are finally broken.

For DNA strand separation in equilibrium, it has been shown that the required work (*F* × *x*) does not depend on the pulling geometry. On this basis, it can be directly explained why higher forces are required for strand separation in the shear geometry, where the length increase during strand separation is smaller.^32^,^40^,^61^ For all three coiled coils measured we detect higher forces (20–50 pN) in the shear geometry than what was previously measured for the unzip geometry. An inverse dependence between the length increase and the magnitude of the force plateau could therefore also hold for coiled coils. From our experimental data, we are not able to determine the equilibrium force in the phase II plateau, thus a direct comparison with published unzipping forces cannot be performed. Considering the superhelical structure of the coiled coil, however, we predict that this simple relationship may not be valid for coiled coils. When mechanically loaded in the unzip geometry, the individual helices are uncoiled at the same terminus. In contrast, in the shear geometry, each mechanically loaded terminus interacts with intact helical structure, experiencing a stabilizing effect. This hypothesis is supported by simulation results of higher order coiled coil oligomers,^21^,^26^ which show higher phase II plateau forces, even though the fundamental helix uncoiling mechanism was observed to be the same. It has further been shown that single helices are stabilized against mechanical uncoiling when interacting with a binding partner.^54^,^62^ To verify this prediction, experiments with longer coiled coils are required, where the phase II plateau can be resolved.

Overall, we have established that shearing occurs shearing occurs out of equilibrium and that the coiled coil length can be used as a parameter for tuning the rupture force of coiled coils. With this new piece of information, we conclude that the mechanical response of coiled coils reproduces many of the essential features of DNA. When loaded in a shear or tensile geometry, coiled coils exhibit an unfolding transition (phase II), highly similar to the overstretching transition in DNA (Fig. 7B).^14^ For short sequences (3–4 heptads), which rupture at forces below or just at the plateau force, dissociation is the dominant strand separation mechanism, so that length can be used as a parameter to tune the rupture force in experiments. For long, natural coiled coil sequences an α-helix to β-sheet transition has frequently been observed in phase III^25^ and SMD simulations predict that this α–β transition should also occur in short coiled coils with a critical minimum length of 4–6 heptads, depending on the loading rate.^27^ Due to the lack of suitable experimental model systems it has so far not been possible to investigate the structural details of this α–β transition. Considering the complexity of the overstretching transition of DNA, where strand separation at loose ends co-exists with melting bubble and S-DNA formation,^32^–^36^ it will be highly interesting to determine the molecular parameters influencing the α–β transition in coiled coils. The coiled coil model system introduced here represents an important starting point towards investigating the sequence–structure–mechanics relationship of coiled coils in a sequence-resolved fashion.

## Conclusion

Using a combination of AFM-based SMFS and SMD simulations, we have investigated the mechanical response of coiled coils of different lengths to an applied shear force. The SMD simulations show that the force first rises almost linearly with extension, before reaching a plateau. The onset of this plateau phase correlates with the uncoiling of helical turns. Combining the SMFS and SMD results suggests that coiled coil strand separation in shear geometry is non-cooperative and follows a hierarchy of timescales: helix uncoiling events are more frequent than sliding events, and sliding events are more frequent than dissociation events. In the simulations, strand separation occurs by uncoiling-assisted sliding in the direction of the applied external force. Dissociation perpendicular to the force axis is not observed in the simulations, suggesting that its intrinsic timescale is much longer than the simulation timescale. Even though strand separation *via* sliding cannot be fully excluded in the experiment, it appears unlikely for the relatively short coiled coils investigated here. For longer coiled coils uncoiling-assisted sliding and dissociation most likely coexist and compete with each other, with a different relative contribution of both mechanisms at different loading rates.

From an application point of view, we have shown that the rupture forces of short dimeric coiled coils are sensitive to the coiled coil length, when mechanically loaded in the shear geometry. Our results represent an important starting point for future experiments aimed at tuning the mechanical stability of coiled coils. Strand separation by either sliding or dissociation is initiated by helix uncoiling, which implies that altering helix stability will directly affect the structural response of coiled coils to shear forces. This may be achieved by introducing helix (de)stabilizing mutations or when using higher order oligomers. Combined with different pulling geometries, a large range of rupture forces can possibly be obtained so that coiled coils have the potential to replace DNA oligonucleotides as nanomechanical building blocks in applications where protein-based structures are desired, *e.g.* as mechanoresponsive material crosslinks^5^,^44^,^45^ or as molecular force sensors.^63^–^65^

## Experimental section

### Peptides

The coiled coil-forming peptides were synthesized using standard solid phase peptide synthesis (SPPS) protocols and obtained from a commercial supplier (Centic Biotec). The peptides were dissolved in coupling buffer (50 mM sodium phosphate pH 7.2 @ 4 °C, 50 mM NaCl, 10 mM EDTA) in a concentration of 2 mM. These peptide stock solutions were aliquoted and stored at –20 °C. For the SMFS experiments, a cysteine was introduced during SPPS at the respective terminus. Peptides without cysteine were used for circular dichroism (CD) spectroscopy to determine the secondary structure and the thermal stability of the three different coiled coils (see ESI†).

### Preparation of glass slides and cantilevers for the AFM measurements

The cysteine-terminated peptides were immobilized to glass slides and AFM cantilevers *via* poly(ethylene glycol) (PEG) spacers, using a previously established protocol.^49^ The A peptide was immobilized to the glass slide, whereas the different B peptides were immobilized to the cantilever. In detail, commercially available, amino-functionalized glass slides (Slide A, Nexterion) were used as amino-functionalized substrates. The cantilevers (MLCT, Bruker) were activated *via* 10 min UV-ozone cleaning and amino-modified using 3-aminopropyl dimethylethoxy silane (ABCR). For the following steps, both glass slides and cantilevers were treated in parallel. Both surfaces were incubated in 50 mM sodium borate (pH 8.5) to increase the fraction of deprotonated amino groups for the subsequent coupling of the heterobifunctional NHS-PEG-maleimide spacer (*M*_W_ = 10 000 g mol^–1^; Rapp Polymere). NHS-PEG-maleimide was dissolved in a concentration of 50 mM in sodium borate and incubated on the surfaces for 1 h. Following incubation, the surfaces were washed with ultrapure water and dried under nitrogen flow. The different B peptides (300 μM in coupling buffer) were pipetted onto the cantilever and incubated on the surface for 1 h at 4 °C. In parallel, a 1 mM solution of the A peptide was incubated on the glass slide. After incubation, the surfaces were rinsed with PBS (10 mM Na_2_HPO_4_, 1.8 mM KH_2_PO_4_, 137 mM NaCl, 2.7 mM KCl, pH 7.4) to remove non-covalently bound peptides and stored in PBS until use.

### Single-molecule force spectroscopy

All SMFS measurements were performed with a ForceRobot® 300 instrument (JPK Instruments) at room temperature in PBS. Cantilever C with a nominal spring constant of 0.01 N m^–1^ was used for all measurements. Each cantilever was calibrated using the thermal noise method^66^ and the spring constants determined varied between 0.016–0.023 N m^–1^. During each experiment, the approach and retract speeds were held constant, and the applied force was adjusted by changing the distance between the tip and the surface. For each sample, several hundreds of approach–retract cycles were carried out on a 10 × 10 μm^2^ grid. To obtain measurements over a broad range of different loading rates, several experiments were performed, each at a different retract speed ranging from 50 to 5000 nm s^–1^. For all three coiled coils, these dynamic SMFS measurements were carried out in three independent experiments, using different cantilevers and glass slides for every experiment.

### Data extraction and analysis

The obtained data was converted into force–extension curves using the JPK data analysis program. The coiled coils were coupled to the surface and the cantilever *via* PEG spacers with a length of approx. 80 nm each (*M*_W_ = 10 000 g mol^–1^). PEG represents an ideal spacer for SMFS measurements. It prevents non-specific binding and possesses a characteristic force–extension behaviour, which can be described with the worm-like chain model (WLC; eqn (1)) at forces below 100 pN:^67^1
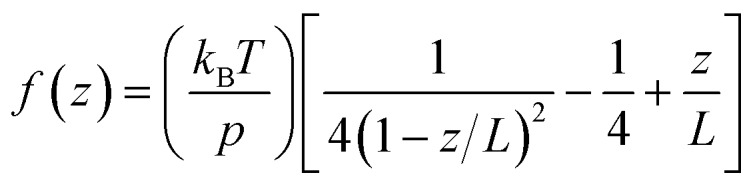
where *f*(*z*) is the force at an end-to-end distance of *z*, *p* is the persistence length, *k*_B_ is the Boltzmann constant, *T* is the absolute temperature, and *L* is the contour length of the polymer (PEG) being stretched.

All force extension curves were fitted with the WLC model. Only force–extension curves that were fitted well (visual inspection) and possessed a contour length >100 nm were used for further analysis. For the selected force–extension curves the rupture forces and the corresponding loading rates were determined using the JPK data analysis program. For each retract speed, the rupture forces and the corresponding loading rates were plotted into histograms (Fig. S3–S5†). All rupture force and loading rate (plotted logarithmically) histograms were fitted with a Gaussian distribution to determine the maxima, which represent the most probable rupture force *F* and the most probable loading rate **, respectively (Table S2†). The most probable rupture forces were plotted against the most probable loading rates and the data was fitted with the Bell–Evans model (eqn (2)):^50^2
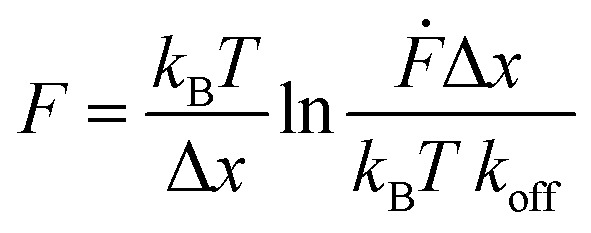
where *k*_B_ is the Boltzmann constant, *T* the temperature, Δ*x* the potential width, *k*_off_ the force-free dissociation rate at zero force, and **, equal to d*F*/d*t*, is the loading rate.

Fitting the *F vs.* ln ** plot with eqn (2), yields *k*_off_ and Δ*x* of the coiled coil interaction. Each data set obtained from one cantilever was fitted separately. The individual values and their corresponding mean ± SEM values are reported in Table S3.†

### Steered molecular dynamics simulations

All simulations were performed using the GROMACS-4.6.7 software package^68^–^71^ and the Amber99-SB force field with the GBSA implicit water model.^72^,^73^ The standard leap-frog integrator for stochastic dynamics with a time step of 2 fs was used. Non-periodic simulation boxes without pressure coupling were used for all simulations in implicit solvent. The Coulomb interaction cut-off was 5 nm, which is sufficiently long for non-periodic simulations. All covalent bonds to hydrogen were constrained to remove fast vibrations. For the explicit solvent simulations, the TIP3P water model was used with periodic boundary conditions. The simulation box size was 17 × 7 × 7 nm^3^ and a NVT ensemble was used during pulling. The fast smooth Particle-Mesh Ewald (PME) method was used to calculate long-range Coulomb interactions and the cut-off for short-range interactions was 1.0 nm. The points of force application were located on the α-C-atoms of the terminal glycines, using virtual harmonic springs with an elastic constant of *k* = 1000 kJ mol^–1^ nm^–2^ = 1650 pN nm^–1^. The distal end of the virtual spring at the N-terminus of peptide A was fixed, while the distal end of the virtual spring coupled to the C-terminus of the B-peptides was moved at a constant speed, parallel to the axis of the coiled coil. All simulations were carried out at room temperature (*T* = 300 K) and the retract speeds varied from *v* = 10^6^ nm s^–1^ to *v* = 10^9^ nm s^–1^. During each constant velocity simulation, the extension Δ*L* = *v* × *t*, where *v* is the retract speed and *t* is time, and the corresponding force *F* felt by the pulled virtual spring were calculated. Simulations of a closely related coiled coil structure^74^,^75^ performed with different spring constants (*k* = 165 pN nm^–1^*vs. k* = 1650 pN nm^–1^) showed a highly similar force–extension behaviour, with the only difference that force fluctuations showed a larger amplitude when the stiffer spring was used (Fig. S11†).

For the coiled coil sequences used no crystal structures are available. Therefore, the initial structures of all coiled coils were generated in the following way: (1) two separate α-helices were generated using the given sequences using Avogadro^48^ and geometry optimized. (2) The individual α-helices were moved together slowly at *T* = 10 K with paired distance restraints between the heptads of each helix. (3) All restraints were removed and the structure was relaxed at *T* = 300 K for 300 ns. The resulting structures were stable at 300 K and showed a typical coiled coil structure, characterized by a left-handed superhelix with paired salt-bridges and a well-defined hydrophobic core. For the two slowest retract speeds, 5 (CC-A_4_B_3_) and 6 (CC-A_4_B_4_) independent simulations were performed in implicit solvent. 20 independent simulations were carried out for both coiled coils at *v* = 10^8^ nm s^–1^. At *v* = 10^9^ nm s^–1^, 40 simulations were performed for CC-A_4_B_4_ and 20 for CC-A_4_B_3_. Only 20 independent runs were performed for CC-A_4_B_3_ at *v* = 10^9^ nm s^–1^, because the comparison of the results from 20 and 40 simulations for CC-A_4_B_4_ at the same retract speed indicated that the lower number of simulations was sufficient to obtain meaningful results. CC-A_4_B_4_ was simulated also in explicit solvent, with 5, 5, 10, and 10 independent runs being performed for the retract speeds of 10^6^, 10^7^, 10^8^ and 10^9^ nm s^–1^, respectively. Each independent run used the same simulation parameters, but started from different initial structures, obtained at different time points of the equilibration simulation (between 200 and 300 ns).

## Conflicts of interest

There are no conflicts of interest to declare.

## Supplementary Material

Supplementary movie

Supplementary movie

Supplementary information
