# Supplementary material for: Molecular mechanics of coiled coils loaded in the shear geometry
Source: Chem Sci. 2018 Apr 23;9(20):4610–21. doi: 10.1039/c8sc01037d (PMC5969510; doi:10.1039/c8sc01037d)
Supplement: Supplementary file 3 [file SC-009-C8SC01037D-s003.pdf]

Electronic Supplementary Material (ESI) for Chemical Science.
This journal is © The Royal Society of Chemistry 2018

Supporting Information

Molecular Mechanics of Coiled Coils Loaded in the Shear Geometry

Melis Goktas,[a,#] Chuanfu Luo,[b,#] Ruby May A. Sullan,[a,§] Ana E. Bergues-Pupo,[b] Reinhard Lipowsky,[b] Ana Vila Verde,[b]* Kerstin G. Blank[a]*

[a]Max Planck Institute of Colloids and Interfaces, Mechano(bio)chemistry, Science Park Potsdam-Golm, 14424 Potsdam, Germany

[b]Max Planck Institute of Colloids and Interfaces, Department of Theory & Bio-Systems, Science Park Potsdam-Golm, 14424 Potsdam, Germany

[#]these authors contributed equally

[§]present address: Ruby May A. Sullan, University of Toronto Scarborough, Department of Physical and Environmental Sciences, 1265 Military Trail, Toronto, M1C 1A4, Canada.

*corresponding authors: Ana.Vilaverde@mpikg.mpg.de; Kerstin.Blank@mpikg.mpg.de

Table of Contents

1. CD spectroscopy of the coiled coils and the corresponding PEG conjugates S3

2. Dynamic single-molecule force spectroscopy of the coiled coils S5

2.1. Force and loading rate histograms of CC-A_4B_4 .... S5

2.2. Force and loading rate histograms of CC-$A_4B_{3.5}$ S6

2.3. Force and loading rate histograms of CC-A_4B_3 .... S7

2.4. Results of the Bell-Evans fits for all 3 cantilevers S9

3. Steered molecular dynamics simulations of the coiled coils S10

3.1. Comparison of explicit and implicit solvent MD simulations S10

3.2. Force vs. strain graphs S11

3.3. Force-extension behaviour of the 6-heptad coiled coil CC-A_6B_6 .... S12

3.4. Coiled coil and α-helix deformation under shear at the slowest retract speed S12

3.5. Influence of the spring stiffness on the force-extension behaviour S16

1. CD spectroscopy of the coiled coils and the corresponding PEG conjugates

The individual cysteine-free peptides were diluted to a concentration of 100 µM in phosphate buffered saline (PBS; 10 mM Na_2HPO_4, 1.8 mM KH_2PO_4, 137 mM NaCl, 2.7 mM KCl, pH 7.4) and subsequently mixed in a 1:1 ratio. To determine a possible effect of the poly(ethylene glycol) (PEG) linker on the secondary structure and the thermal stability of the coiled coil, PEG conjugates were investigated in addition. For this purpose, methoxy-PEG-maleimide (MW = 10000 g mol^{-1}; Rapp Polymere) was reacted with the individual cysteine-containing peptides in an 1:1 ratio (20 °C, 300 rpm, 15 min) before mixing the peptides for the CD measurement.

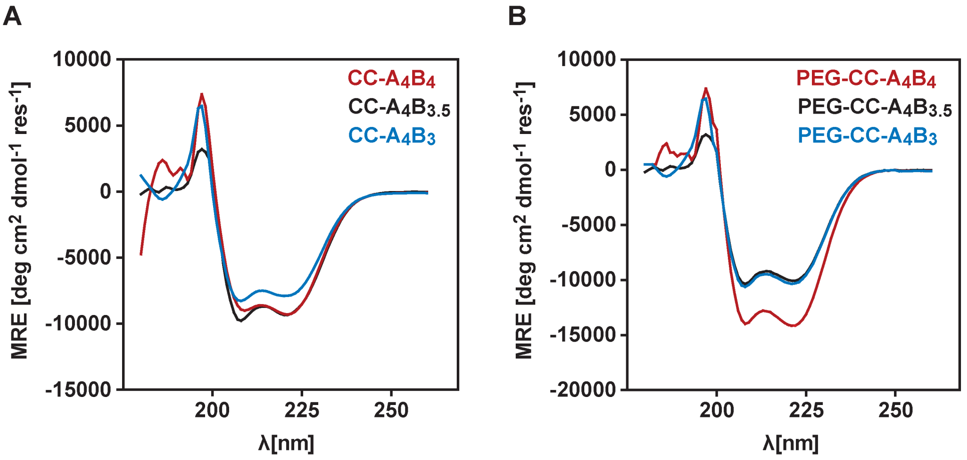

Figure S1. CD spectra of the coiled coils and the corresponding PEG conjugates (20°C). A) Comparison of the CD spectra of the coiled coils (cysteine-free peptides). B) Comparison of the CD spectra of the coiled coil-PEG conjugates (10000 g mol^{-1} PEG coupled to the N-terminal cysteine of A_4 and the C-terminal cysteine of B_4, $B_{3.5}$ and B_3, respectively). All measurements were performed in PBS, using a total peptide concentration of 100 µM.

CD spectra and thermal denaturation curves were recorded in 1 mm quartz cuvettes (Hellma Analytics) using a Chirascan CD spectrometer (Applied Photophysics) equipped with a Peltier temperature controller. For the CD spectra, the samples were measured at 20 °C, using the following settings: 0.7 s time-per-point, 1 nm step size and 1 nm bandwidth (Figure S1). Thermal denaturation experiments were performed under the same conditions, heating the

samples from 5 to 90 °C at a rate of 1 °C min^{-1}. The CD signal at 222 nm was recorded using the same settings (Figure S2). The melting temperature T_m was determined from the second derivative, *i.e.* the change point, of the thermal denaturation curves. The results are summarized in Table S1. Each measurement was performed in triplicate. The errors given represent the standard error of the mean (SEM).

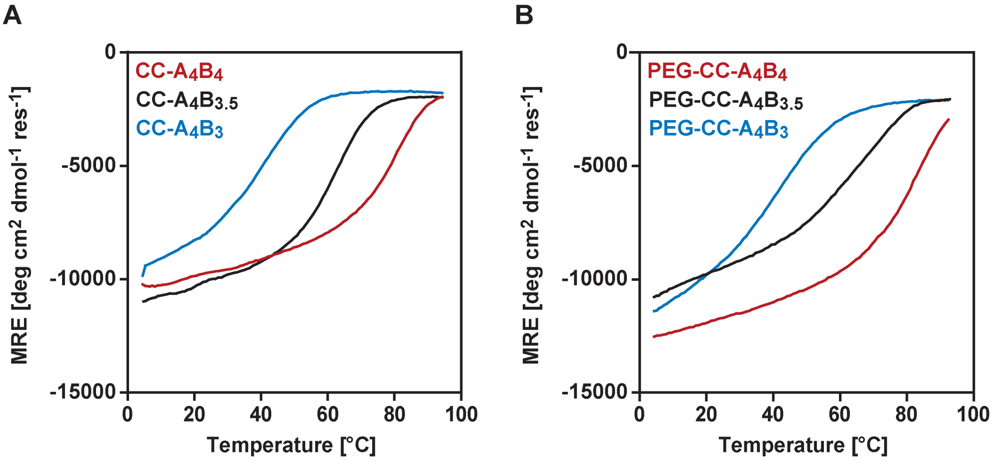

Figure S2. Thermal denaturation of the coiled coils and the corresponding PEG conjugates (222 nm). A) Thermal stability of all coiled coils without terminal cysteines. B) Thermal stability of the coiled coil-PEG conjugates (10000 g mol^{-1} PEG coupled to the N-terminal cysteine of A_4 and the C-terminal cysteine of the respective B peptide). All measurements were performed in PBS, using a total peptide concentration of 100 µM.

Table S1. Summary of the melting temperatures for the three different coiled coils (T_{m_CC}) and the corresponding PEG conjugates ($T_{m\_CC\text{-}PEG}$). The data represents the mean ± SEM of 3 independent measurements.

**heterodimer**	T_{m_CC} **[°C]**	$T_{m\_CC\text{-}PEG}$ **[°C]**
CC-A_4B_4	80.5 ± 0.8	79.9 ± 0.1
CC-$A_4B_{3.5}$	61.0 ± 0.1	64.9 ± 0.3
CC-A_4B_3	39.0 ± 0.1	39.4 ± 0.3

2. Dynamic single-molecule force spectroscopy of the coiled coils

2.1. Force and loading rate histograms of CC-A_4B_4

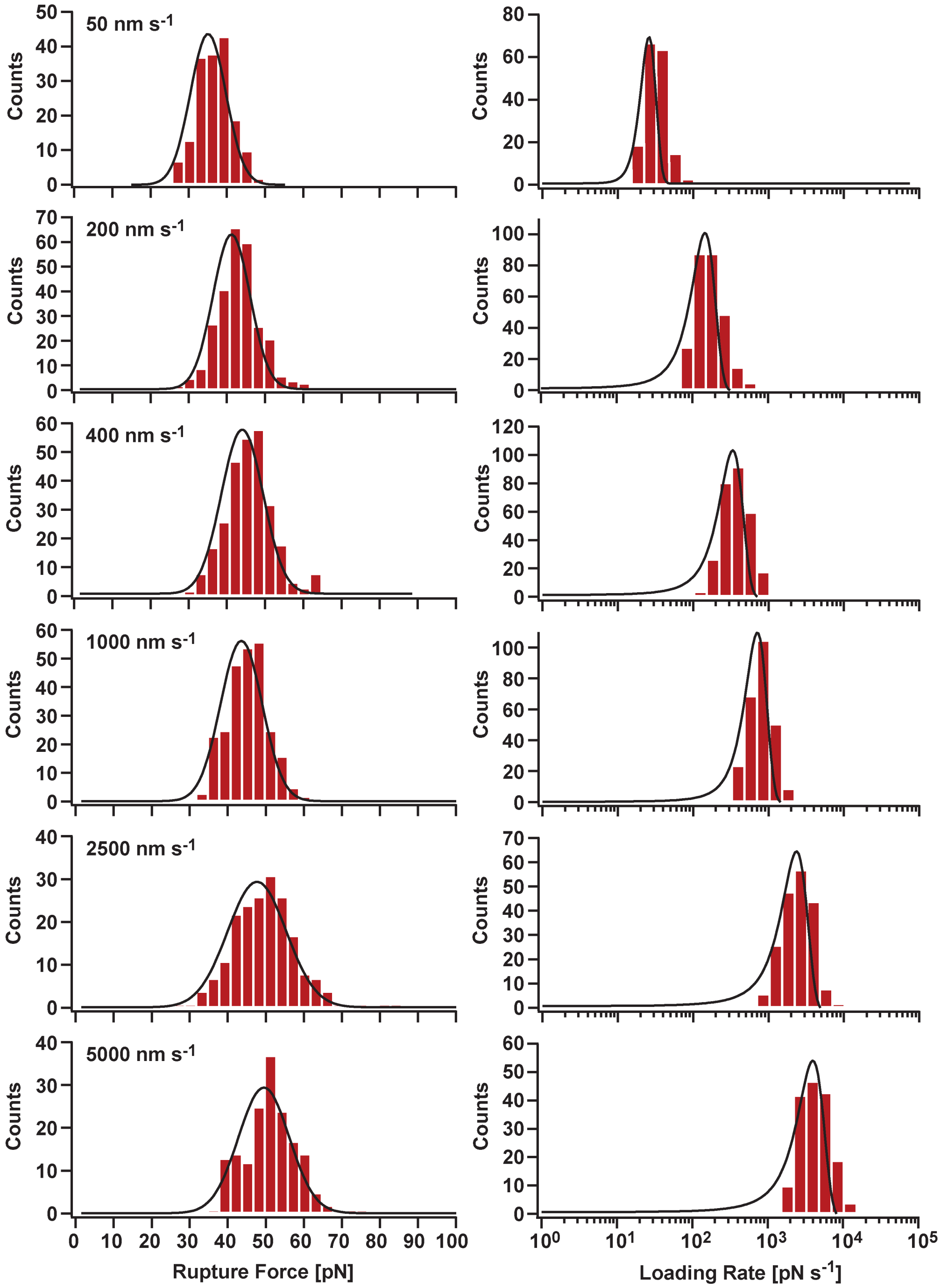

Figure S3. Example data set of CC-A_4B_4 measured at 6 different retract speeds. The black lines represent Gaussian fits to the data. Even though the Gaussian fit does not describe the underlying physical process, it represents the distributions sufficiently well to allow for extracting the most probable rupture forces and loading rates.

2.2. Force and loading rate histograms of CC-$A_4B_{3.5}$

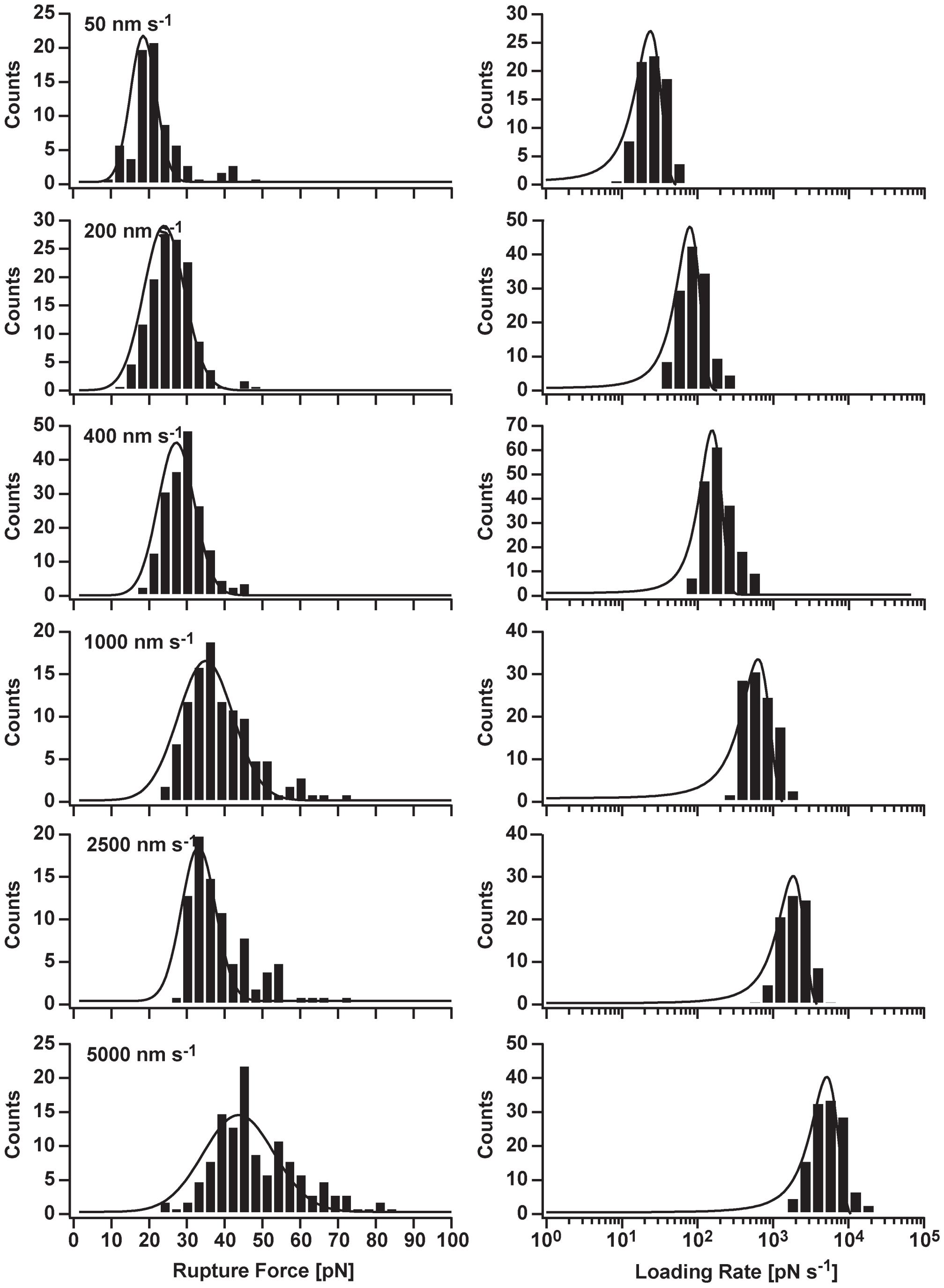

Figure S4. Example data set of CC-$A_4B_{3.5}$ measured at 6 different retract speeds. The black lines represent Gaussian fits to the data. Even though the Gaussian fit does not describe the underlying physical process, it represents the distributions sufficiently well to allow for extracting the most probable rupture forces and loading rates.

2.3. Force and loading rate histograms of CC-A_4B_3

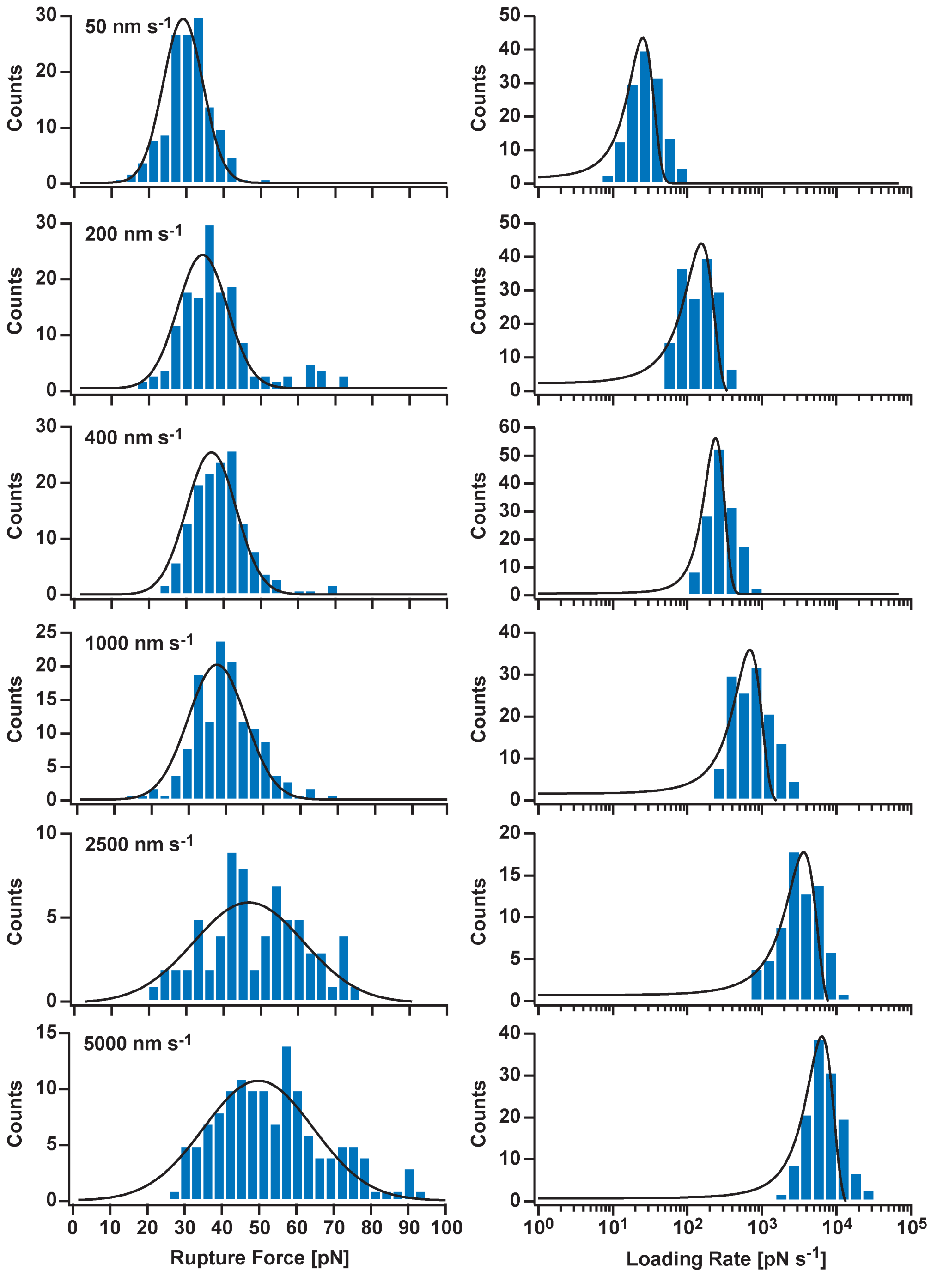

Figure S5. Example data set of CC-A_4B_3 measured at 6 different retract speeds. The black lines represent Gaussian fits to the data. Even though the Gaussian fit does not describe the underlying physical process, it represents the distributions sufficiently well to allow for extracting the most probable rupture forces and loading rates.

Table S2. Summary of the rupture forces F and loading rates $\dot{F}$ obtained from Gaussian fits to the respective distributions.

		cantilever 1			cantilever 2			cantilever 3		
**CC**	v [nm s^{-1}]	n^a	F [pN]	$\dot{F}$ [pN s^{-1}]	n^a	F [pN]	$\dot{F}$ [pN s^{-1}]	n^a	F [pN]	$\dot{F}$ [pN s^{-1}]
**CC-A_4B_4**	50	170	35.1	27	201	34.2	27	84	33.2	30
	200	273	41.2	147	168	40.8	117	224	42.3	117
	400	285	44.0	335	136	43.8	236	294	39.0	237
	1000	260	43.6	711	139	46.4	547	240	47.4	764
	2500	193	47.7	2351	105	48.8	1530	224	48.7	1678
	5000	166	49.5	3972	159	52.0	4807	185	52.7	4987
**CC-$A_4B_{3.5}$**	50	77	18.5	24	74	19.6	35	---	---	---
	200	133	23.9	79	86	30.0	252	103	26.2	86
	400	187	27.2	158	92	29.6	647	98	27.8	170
	1000	108	34.9	631	144	37.8	974	110	32.5	487
	2500	88	33.1	1846	119	39.1	2159	138	36.7	1593
	5000	127	43.7	5141	82	48.1	7267	155	45.3	5795
**CC-A_4B_3**	50	139	29.1	25	152	31.5	31	65	33.3	46
	200	158	34.4	155	137	36.2	265	77	33.7	151
	400	145	36.7	239	105	35.4	381	94	39.7	360
	1000	136	38.2	591	172	38.3	769	131	43.5	1058
	2500	70	46.7	3636	139	43.1	2070	174	45.8	2975
	5000	133	49.8	6406	184	56.4	7364	111	53.3	6059

[a] *n* represents the number of force curves analysed

2.4. Results of the Bell-Evans fits for all 3 cantilevers

For every coiled coil, 3 independent experiments were performed using different cantilevers and surfaces. Each data set was fitted independently with the Bell-Evans model to obtain the corresponding k_{off_SMFS} and Δx_{SMFS} values (Table S3). Using these values, the mean ± SEM were determined.

Table S3. Summary of the k_{off_SMFS} and Δx_{SMFS} values for the different coiled-coils CC-A_4B_4, CC-$A_4B_{3.5}$ and CC-A_4B_3 obtained from Bell-Evans fits of 3 independent experiments. The table also shows the corresponding mean ± SEM.

	**individual measurements**		**mean values**	
**CC**	$\boldsymbol{k_{off}}$ **[s^{-1}]**	**Δx [nm]**	$\boldsymbol{k_{off}}$ **[s^{-1}]**	**Δx [nm]**
**CC-A_4B_4**	1.8×10^{-5}<br>2.2×10^{-4}<br>7.3×10^{-4}	1.52<br>1.22<br>1.12	$(3.2 \pm 2.1) \times 10^{-4}$	1.29 ± 0.12
**CC-$A_4B_{3.5}$**	6.8×10^{-2}<br>1.9×10^{-1}<br>6.9×10^{-2}	0.96<br>0.79<br>0.92	$(1.1 \pm 0.4) \times 10^{-1}$	0.89 ± 0.05
**CC-A_4B_3**	3.6×10^{-3}<br>1.1×10^{-2}<br>4.7×10^{-3}	1.10<br>0.96<br>1.03	$(6.5 \pm 2.4) \times 10^{-3}$	1.03 ± 0.04

3. Steered molecular dynamics simulations of the coiled coils

3.1. Comparison of explicit and implicit solvent MD simulations

To assess whether implicit solvent simulations bias the response of coiled coils to shear loads, we performed explicit solvent simulations with CC-A_4B_4 at the same 4 retract speeds also considered in the implicit solvent simulations. The results (Figure S6) of the implicit and explicit solvent simulations share very similar features: in both cases, the force-extension curve is characterized by an initial rise phase, followed by a constant force plateau and finally a zero force phase, indicating coiled coil separation. The transitions between the three phases occur at similar extensions for implicit and explicit solvent simulations. The plateau forces are circa 20 % lower in explicit solvent, when compared to the implicit solvent simulations. This similarity suggests that the molecular processes responsible for the constant force plateau are similar in both types of simulations.

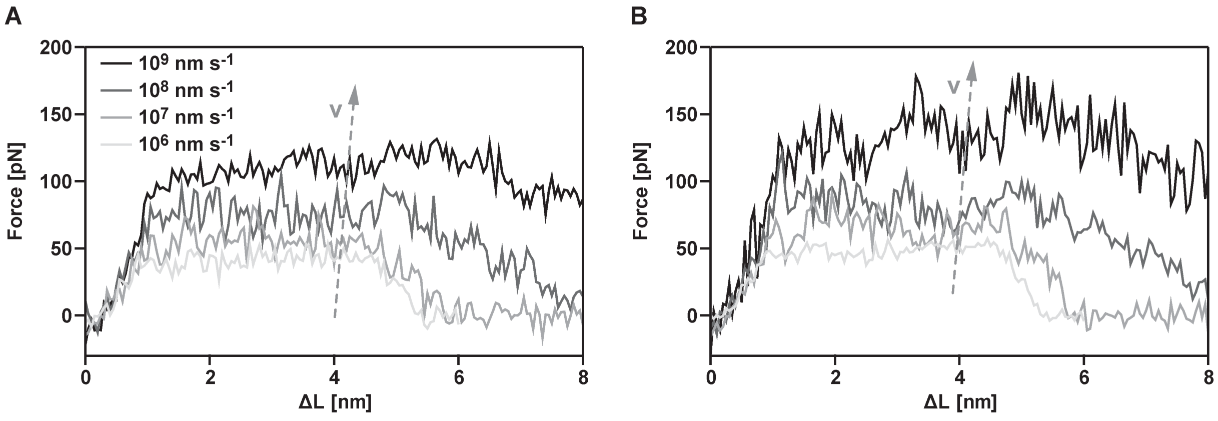

Figure S6. Comparison of the force-extension (ΔL) curves from simulations using A) explicit and B) implicit water models (CC-A_4B_4). The results of the simulations in explicit water are averaged from 10 (10^9 nm s^{-1}, 10^8 nm s^{-1}) or 5 (10^7 nm s^{-1}, 10^6 nm s^{-1}) independent runs, respectively. The results of the simulations in implicit water are averaged from 40 (10^9 nm s^{-1}), 20 (10^8 nm s^{-1}) and 6 (10^7 nm s^{-1}, 10^6 nm s^{-1}) independent runs.

3.2. Force vs. strain graphs

To be able to compare the response of the coiled coils studied here with earlier simulations of natural coiled coils, we have converted the extension (ΔL) into strain ($\Delta L/L_0$) and replotted Figure 5 in the main text (Figure S7). The equilibrium length L_0 was obtained from force-free simulations of a duration of 200 ps. L_0 equals 4.21 nm for CC-A_4B_4 and 3.12 nm for CC-A_4B_3. The force vs. strain curves for CC-A_4B_4 and CC-A_4B_3 show that the phase I→II transition occurs between 15 and 25 % strain, thereby matching the results obtained for other coiled coils.

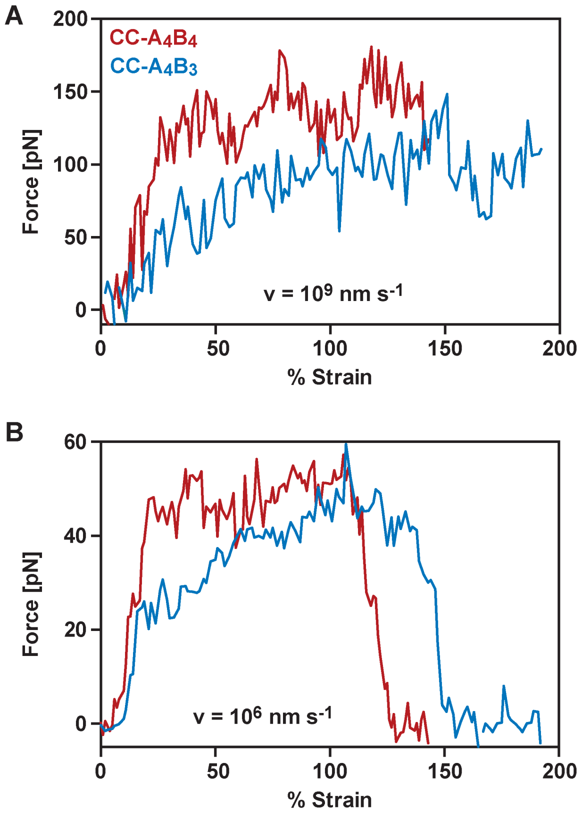

Figure S7. Averaged force-strain curves of the different coiled coils obtained from SMD simulations (T = 300 K, implicit solvent). The graph shows the forces as a function of strain ($\Delta L/L_0$). A) Force-strain behaviour at the fastest retract speed ($v = 10^9$ nm s^{-1}). The results are averaged from 20 (CC-A_4B_3) and 40 (CC-A_4B_4) independent runs. B) Force-strain behaviour at the slowest retract speed ($v = 10^6$ nm s^{-1}). The results are averaged from 5 (CC-A_4B_3) and 6 (CC-A_4B_4) independent runs.

3.3. Force-extension behaviour of the 6-heptad coiled coil CC-A_6B_6

To determine a possible effect of further extending the length of the coiled coil, preliminary simulations were performed with a 6-heptad long sequence, CC-A_6B_6, formed by repeating the two N-terminal heptads of CC-A_4B_4. The coiled coil sequence is A6 - G EIAALEQ EIAALEK EIAALEQ EIAALEK ENAALEW EIAALEQ G and B6 - G KIAALKQ KIAALKY KIAALKQ KIAALKY KNAALKK KIAALKQ G. The general force extension-behaviour of CC-A_6B_6 is very similar to the other coiled coils investigated (Figure S8); however, the average plateau force (84 pN) is higher than for the CC-A_4B_4 and CC-A_4B_3 simulated at the same retract speed.

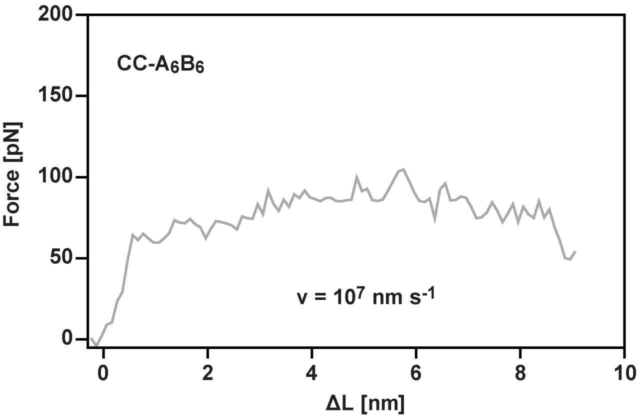

Figure S8. Averaged force-extension curve for coiled coil CC-A_6B_6 obtained from SMD simulations (T = 300 K; implicit solvent). The graph shows the force as a function of extension ($\Delta L = v \cdot t$, where v is the retract speed and t is time) for a retract speed of $v = 10^7$ nm s^{-1}. The result represents the average over 6 independent simulation runs. The average force over an extension interval of [2<ΔL<6] is 84 pN, with an associated standard error of the mean of 1 pN.

3.4. Coiled coil and α-helix deformation under shear at the slowest retract speed

To further characterize the unfolding-assisted sliding mechanism, we have calculated multiple intermolecular and intramolecular distances for pairs of selected amino acids. These distances give insight into the molecular process of rupture and reformation of interchain contacts. At the

same time, this analysis shows how these processes determine local helix uncoiling and recoiling, as a function of CC elongation. Figure S9 shows the interhelical distances between 4 selected pairs of interhelical salt bridges (Figure S9A) or hydrophobic contacts (Figure S9B) in CC-A_4B_4 during a single simulation run performed at the slowest retract speed ($v = 10^6$ nm s^{-1}). These two plots clearly show a step-wise interhelical displacement, with a step size of approximately 1 nm. This step size is consistent with the length of 1 heptad (7×0.15 nm = 1.05 nm). This is a clear indication that the original salt bridges and hydrophobic contacts break and are immediately replaced with new ones, if neighbouring partners are still available. Despite the step-wise nature of sliding, we never observe the formation of intermediate states where most of the salt bridges and coiled coils are broken. One could naively assume that these states would form if the two α-helices were displaced by approximately 0.5 nm, *i.e.*, if the helices were maximally out-of-register. Figures S9A and S9B, however, clearly show that these states do not form.

To characterize local helix uncoiling and recoiling, we have further analysed the intrahelical distances of selected pairs of amino acids within α-helix A (Figure S9C) and B (Figure S9D) at the slowest retract speed ($v = 10^6$ nm s^{-1}). The amino acid pairs chosen are separated by 3 amino acids (*i*, *i*+4 pairs). At zero elongation, the amino acids are located on the same face of the helix and correspond to one folded helical turn. Large deviations from their initial distance signal the opening of helical turns. Figures S9C and S9D show that some turns of each helix open transiently, but the helices reform after some time. Importantly, recoiling roughly coincides with the position of the steps observed in the distances determined for the salt bridges and hydrophobic contacts, *i.e.* at $\Delta L \approx 2, 3, 4$ nm. These results indicate that local helix uncoiling and recoiling facilitates the sliding of the two helices under shear load. The observed local deformation of the helices enables sliding to occur without the high energetic cost associated with simultaneously breaking all hydrophobic contacts and salt bridges.

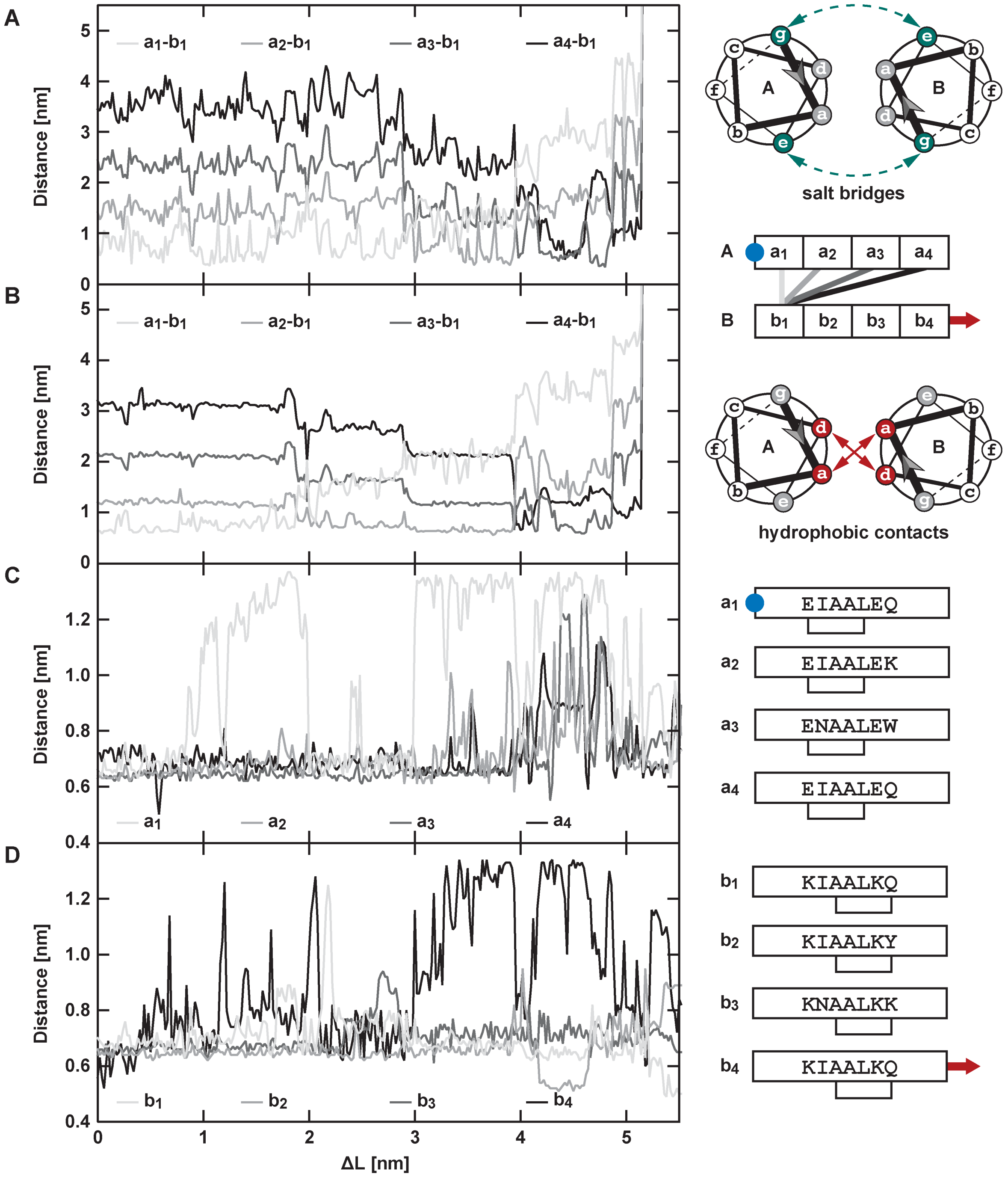

Figure S9. Intermolecular distances characterizing the rupture and reformation of interhelical contacts and intramolecular distances characterizing the deformation of the individual helices (CC-A_4B_4). The analysis has been performed for a single implicit solvent simulation run at the slowest retract speed ($v = 10^6$ nm s^{-1}). A) Interhelical distances of 4 selected salt bridge pairs, as illustrated in the cartoon on the right-hand side. Plotted are the shortest distances between the side chain O- and N-atoms of the charged glutamic acid (*g* positions in heptads a_1-a_4) and lysine (*g* position in b_1) residues in the indicated heptads. a_i - b_j denotes the pair between the

i-th heptad of α-helix A and the *j*-th heptad of α-helix B. The blue circle and the red arrow denote the fixed and retracted end, respectively. B) Interhelical distances of 4 selected hydrophobic contacts, as illustrated in the cartoon on the right-hand side. Plotted are the distances between the centres of mass of the amino acids in the *a* positions of heptads a_1-a_4 and the *a* position of b_1. (C, D) Intrahelical distances of 4 selected pairs of α-carbons separated by 3 amino acids in both α-helices (*i*, *i*+4). The selected amino acids are illustrated in the cartoon on the right-hand side.

To assess whether the transient helix uncoiling observed at the slowest retract speeds reflects the uncoiling of full helical turns, we have calculated the intrahelical distances for pairs of amino acids within one helical turn. Figure S10 shows these distances for selected pairs of amino acids separated by only one amino acid (*i, i+2* pairs). The results indicate that consecutive *i, i+2* pairs often uncoil separately and that the uncoiling of helical stretches shorter than full helical turns is a frequent event.

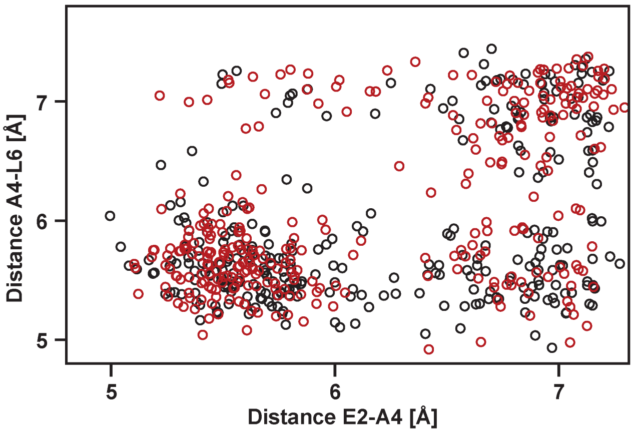

Figure S10. Intrahelical distances between the α-carbons of selected *i, i+2* amino acid pairs in the first heptad of α-helix A (CC-A_4B_4). The data points are obtained from the analysis of two different implicit solvent simulations at the slowest retract speed ($v = 10^6$ nm s^{-1}).

3.5. Influence of the spring stiffness on the force-extension behaviour

All simulations described in this manuscript were performed with a spring constant of k = 1000 kJ mol^{-1} nm^{-2} = 1650 pN nm^{-1}. This spring constant is several orders of magnitude higher than that associated with the PEG spacers used in the SMFS experiments. As a consequence, the loading rates in the simulations are also much higher. This difference between experiment and simulation is unavoidable. Using spring constants with k << 1650 pN nm^{-1}, combined with low pulling velocities, would result in simulation times too long to be feasible.

Within computationally accessible limits, our results are qualitatively unaffected by the choice of the spring stiffness. Unpublished simulations of a structurally related trimeric coiled coil with similar length and sequence (initially proposed by Nautiyal et al., Biochemistry, 1995, 34:11645; Nautiyal & Alber, Protein Science, 1999, 8:84) indicate that the mechanisms leading to coiled coil strand separation under shear are the same at our chosen spring constant (k = 1650 pN nm^{-1}) and with k = 165 pN nm^{-1}. This last spring constant value is the lowest that is computationally feasible for the two retract speeds tested (v = 10^7 nm s^{-1} and v = 10^8 nm s^{-1}).

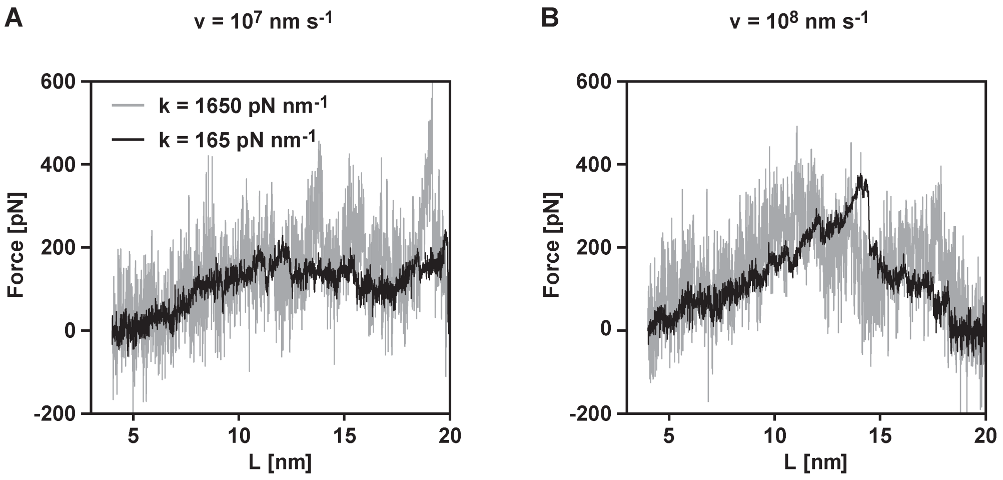

Figure S11. Force-extension curves of a structurally related heterotrimeric coiled coil, simulated using different spring constants. The curves are obtained from single realizations at the indicated spring constant values and retract speeds.

As shown in Figure S11, a larger spring constant increases the magnitude of the force fluctuations at the spring, but does not markedly alter the average value of the force or the characteristics of the force-extension curves: at the lower velocity ($v = 10^7$ nm s^{-1}), an initial rise phase followed by a force plateau is present for both spring constants; at the higher velocity ($v = 10^8$ nm s^{-1}), neither curve displays an easily identifiable plateau region, similarly to single realizations of the dimeric coiled coils. Visual inspection of the trajectories (not shown) confirms that the two spring constants show similar strand separation mechanisms.
